# A chiral microchip laser using anisotropic grating mirrors for single mode emission

**DOI:** 10.1515/nanoph-2022-0783

**Published:** 2023-03-24

**Authors:** Fangfang Li, Shawn Lapointe, Théo Courval, Marina Fetisova, Thomas Kämpfe, Isabelle Verrier, Yves Jourlin, Petri Karvinen, Markku Kuittinen, Jean-François Bisson

**Affiliations:** Center for Photonics Sciences, University of Eastern Finland, P.O. Box 111, FI-80101 Joensuu, Finland; Département de physique et d’astronomie, Université de Moncton, 18 Antonine-Maillet Ave., E1A 3E9, Moncton, Canada; Laboratoire Hubert Curien UMR 5516, Univ. Lyon, UJM-Saint-Etienne, CNRS, Institut d’Optique Graduate School, F-42023, Saint-Etienne, France

**Keywords:** nanostructured mirrors, PT-symmetric lasers, resonant gratings, single mode lasers

## Abstract

A pair of nanostructured mirrors made of a diffraction grating inscribed in the top layer of a Bragg mirror are designed such that a phase shift near π and different reflected amplitudes exist between transverse electric (TE) and magnetic (TM) reflected polarization states at normal incidence. When a standing wave laser resonator is formed with two such mirrors and the two mirrors’ principal axes are twisted one with respect to the other, this phase shift condition suppresses multiple longitudinal mode emission arising from axial spatial hole burning. In addition, the different amplitudes of TE and TM reflected polarizations create polarization eigenstates with different round-trip losses, suppressing one polarization eigenstate. Laser experiments made with a Yb^3+^-doped Y_3_Al_5_O_12_ active material reveal enhanced purity of the emission spectrum compared to similar lasers using conventional laser mirrors. The proposed design enables a miniature single mode laser, replacing more complex designs usually needed to achieve that goal.

## 1 Introduction

Achieving single mode laser emission at high power from a miniature device is key for applications requiring very small spaces, first and foremost in photonic integrated circuits [1] but also in laser-based free-space communications in air- or space-borne platforms [2], light detection and ranging (LiDAR) for the automotive industry [3], and remote sensing in general [4]. This quest has led to the development of vertical-cavity surface-emitting semiconductor lasers (VCSELs) and, more recently, to other original on-chip integrated lasers architectures of various types, such as bound-state-in-a-continuum (BIC) nanolasers [5–7], topological insulator lasers [8–10], parity-time (PT) symmetric lasers [11, 12] and other designs harnessing metasurfaces [13, 14]. Many concepts underlying these lasers were borrowed from condensed matter and quantum physics. They exploit light confinement at the nanoscale as well as Fano-type interferences and the interplay of gain and loss between interacting semiconducting laser microresonators. Importantly, they can be realized with technologies compatible with the well-established planar batch processing of the semiconductor CMOS industry. The laser miniaturization within the CMOS technology should preserve single mode operation, i.e., high temporal and spatial coherence with a high degree of polarization, as well as high power emission with high optical conversion efficiency. Moreover, design robustness and versatility, such as wavelength tunability and polarization switching, are important features for the successful application of these designs.

The device proposed in this paper is based on the concept PT symmetry in the polarization space. Since C. Bender and S. Boettcher’s seminal paper demonstrating that parity-time symmetric operators can exhibit, like Hermitian systems, entirely real eigenvalue spectrum and spontaneous symmetry breaking [15], this topic has attracted considerable attention, especially in optics. Single-mode lasers based on selective parity-time symmetry breaking were demonstrated [16, 17] and, as a result, a new paradigm emerged, that a PT-symmetric laser should be an open system that includes some form of coupling to the exterior. Yet, it was shown that a resonator made of a pair of anisotropic mirrors can display polarization eigenstates that have the PT-symmetric character, without relying on the concept of interplay between gain and loss [18]. The relative orientation, *α*, of the two resonator mirrors can be used as a control parameter that spans regions of unbroken and broken PT symmetry, including the transition at the branch point singularity where the two states are degenerate. The proof of concept was demonstrated by mimicking anisotropic mirrors with intracavity elements placed in front of conventional isotropic mirrors [18]. Although a clear improvement in the purity of the emission spectrum was observed near the exceptional point, the resonator was rather bulky and the long resonator length resulting from the intracavity elements created mode hopping due to the very close spacing between consecutive longitudinal modes.

Here, we report on the single mode emission of a PT-symmetric laser in polarization eigenstates using anisotropic mirrors. The replacement of intracavity bulky elements with nanostructured mirrors enables both miniaturization and simplification of the resonator design by doing away with intracavity bulk elements. The importance of this development stems from the fact that it makes the design compatible with 2D planar batch processing of the semiconductor industry. Since it also allows the miniaturization of the device, we show that the resulting thinner resonator allows stabilization of the emission spectrum by increasing the mode spacing. In addition, the state of polarization is shown to be controllable, with the demonstration of circular polarization states of opposite chiralities by changing the orientation of the mirrors’ principal axes by a few degrees. It also allows efficient cooling without compromising the compactness of the resonator. These aspects open opportunities for integration, power scaling and versatility.

This paper is organized as follows. In Section 2, the concept of a chiral PT-symmetric laser made of two anisotropic mirrors is described. In Section 3, the desired physical characteristics of the two laser mirrors are described and the design of the mirrors is presented. It is followed in Section 4 by a description of the fabrication method and the optical characterization of these mirrors. Because of the strict tolerance on the phase shift between orthogonal polarization directions, ten different grating samples differing by their fill factor (grating linewidth divided by period) were made, to reduce the impact of systematic errors in the parameters. These measurements enabled us to choose the combination of grating mirrors closest to the specifications for the laser experiments. In Section 5, we present laser experiments, with the emphasis put on the polarization eigenstates and eigenvalues, and on the emission spectrum. We also report on the efficiency, degree of polarization and beam quality of the device. This paper is concluded in Section 6 with a discussion and conclusion.

## 2 Theoretical background

Our concept takes its roots in Siegman’s and Evtuhov’s seminal work [19] on the twisted-mode laser. They showed that, by placing quarter-wave plates in front of each mirror of a standing wave laser resonator, the eigenmodes acquire left and right circular polarization states. Importantly, the counter-propagating waves of each mode become mutually orthogonal, which implies that the intra-cavity intensity modulation cancels out. Thus, axial spatial hole burning, which gives rise to multiple longitudinal mode oscillation in homogeneously broadened laser materials, can be eliminated. However, with such a design, the two circular polarization states coexist because they experience similar round-trip losses; by inserting a polarizer between the quarter wave plate and the output coupler [20–22], one circular polarization can be eliminated and dual polarization emission, suppressed.

Now, when using laser mirrors with different attenuation between their two principal axes, in addition to the π phase shift, the resonator acquires a PT-symmetric character [18] and new phenomena take place. First, polarization eigenstates are no longer mutually orthogonal [23, 24] and can merge to a unique eigenstate, called an exceptional point (EP) [25], at some specific relative angle, *α* = *α*_0_, of the two mirrors’ principal axes. An EP marks the transition between a region of *α* values, for *α* < *α*_0_, called the unbroken symmetry region or region I, where eigenvalues of the Jones matrix of a round trip inside the resonator are real, and a second region, for *α* > *α*_0_, called the broken symmetry region or region II, where they are complex conjugate [16, 17]. In region I, the polarization eigenstate with higher loss is easily suppressed while, in region II, the two polarization eigenstates experience equal losses but different round-trip phase shifts and are thus prone to coexist at different emission frequencies. However, the intensity contrast of the standing wave is high in region I but is found to vanish in region II; hence, the consequent flattening of the axial intensity distribution inside the active material in region II eliminates spatial hole burning and single longitudinal mode emission is thus likely to take place.

**Figure 1:**
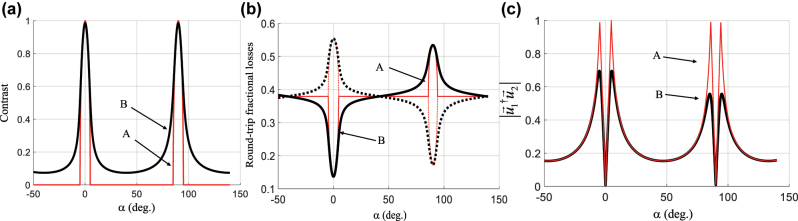
Calculated characteristics arising from the eigenvector analysis of the round-trip Jones matrix. (a) Contrast of the standing wave of each mode (perfectly superimposed on the graph); (b) round-trip fractional losses of each polarization eigenstate (solid and dotted lines respectively); (c) proximity of the two eigenstates, as a function of *α* for two cases shown in Table 1 (red and black for cases A and B, respectively, shown in Table 1).

Now, when there is a discrepancy in the phase shift Δ with respect to the targeted π value, the contrast of the standing wave no longer drops to zero in region II, but the round-trip loss contrast between the two polarization eigenstates remains high in region I. The contrast of the standing wave, the round-trip losses and the orthogonality of the polarization eigenstates are shown in Figure 1(a)–(c) for the ideal case (curve A in red) and for the measured parameter values of the fabricated mirrors (curve B in black), listed in Table 1. For such a device deviating from ideal specifications, the question arises whether there exists an *α* value at which both dual polarization and multiple longitudinal mode emission are suppressed. It will be shown that such region of *α* values exists that realizes the best compromise between efficient suppression of dual polarization emission and suppression of multi-longitudinal emission. A laser design that would tap into the advantages of each region, i.e., the simultaneous suppression of dual polarization emission and of multiple longitudinal mode emission, would be attractive for achieving single mode emission. It would open a new avenue for the generation of narrow bandwidth emission from miniature devices that does not resort to bulky laser architectures such as a unidirectional ring cavity [26], injection seeding [27, 28], master oscillator-power amplifier [29] or active frequency stabilization schemes [30]. It would also appear as an alternative to other more recent nanolasers schemes offered by BICs, topological insulators and other metasurfaces.

**Table 1:** Parameter values used in the simulation shown in Figure 2. Case A is the ideal design, case B corresponds to measured values from fabricated samples.

Case	Pump mirror		Output mirror	
	Δ_1_ (°)	*R*_TE_/*R*_TM_	Δ_2_ (°)	*R*_TE_/*R*_TM_
A (ideal)	180.0	0.97/0.93	180.0	0.89/0.48
B (actual)	178.4		188.8	

## 3 Design of the anisotropic laser mirrors

### 3.1 General considerations

Anisotropic Bragg mirrors at normal incidence require the introduction of form anisotropy; this can be realized with nanostructured optical thin films by using two classes of techniques. The first one is a bottom–up approach wherein artificial anisotropic effective materials are made by glancing angle deposition to produce anisotropic mirrors for laser resonators. These are nanoporous structures, whose morphology is such that the in-plane effective indices have values differing by Δ*n* ≈ 0.1 [31]. Such a concept was demonstrated in ref. [32] with the goal to achieve a twisted-mode laser for eliminating multimode emission.

An alternative approach consists in using nanofabrication techniques for inscribing a one-dimensional periodic grating on top of a multilayer Bragg mirror to create adequate anisotropic optical responses of TE and TM polarization states at normal incidence. The optical response of different polarization states depends on the property of the layers (i.e., refractive index and thickness) and the dimension of the grating (i.e., grating depth, period and fill factor) and this offers the possibility to control both the reflected amplitude of the TE and TM polarizations, i.e., diattenuation, and their phase shift, i.e., diretardance. It is this approach that was followed in this work.

We designed a pair of anisotropic grating mirrors such that a π phase shift and differential amplitudes exist between reflected TE and TM polarization states at the operating wavelength, *λ* = 1030 nm, of a Yb^+3^-doped YAG laser. When a standing wave resonator is formed with two such mirrors, adjusting the relative orientation, *α*, of the two mirrors’ principal axes also enables one to adjust the contrast of the intra-cavity standing wave by changing the proximity of the polarization states of the two counter-propagating waves, as explained in Section 2.

**Figure 2:**
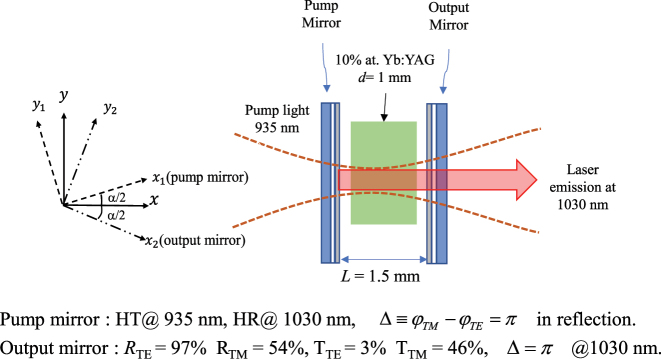
Sketch of the chiral microchip laser equipped with anisotropic mirrors, with their specifications.

The design of the chiral laser resonator is shown in Figure 2. We consider a laser-diode, end-pumped Yb^3+^-doped Y_3_Al_5_O_12_ (YAG) microchip laser equipped with anisotropic mirrors having both diattenuation and diretardance. The ideal characteristics of such mirrors are as follows. The pump mirror is highly transparent (HT) at the pump wavelength (*λ*_p_ = 935 nm). In addition, the pump mirror is highly reflecting (HR) around the emission wavelength, *λ*_l_ = 1030 nm, for both TE and TM polarizations and the phase shift between reflected TE and TM polarizations is π. The output mirror also produces a phase shift of π at *λ*_l_ in reflection, and also diattenuation, with design values $R_{TE}\equiv {\left|r_{TE}\right|}^{2}=97\text{\%}$ and $R_{TM}\equiv {\left|r_{TM}\right|}^{2}=54\text{\%}$ . The latter numerical values are chosen to obtain an EP at the twisting angle *α*_0_ ≈ 5°, which is large enough for easily controlling the transition from region I to region II in an actual experiment.

### 3.2 The determination of the grating parameters

The fundamental idea of the anisotropic mirror produced with a diffraction grating lies in the use of grating-mediated waveguide resonances, which couple light from the grating into the uppermost layers of the multilayer system, which is then coupled back to reflected or transmitted diffraction orders of the grating. This coupling can lead to a significant enhancement or suppression of the base reflection characteristics of the underlying multilayer [33]. In this work, the strong dependence of such waveguide coupling on the polarization of the incoming light is used [34]. This mechanism is often applied to select one polarization in reflection and/or transmission to achieve polarization selective devices, e.g., for intracavity polarization selection [35, 36]. Here, the polarization-dependent complex reflection amplitude is the desired property, wherein the introduction of a π phase shift between TE and TM polarizations is necessary. As is shown in [33], analytical models can predict the coupling coefficients between grating and waveguide modes, showing that they depend strongly on the polarization, in both amplitude and phase. It is therefore justified to look for a grating mirror configuration that fulfills the specifications outlined in Section 3.1.

**Figure 3:**
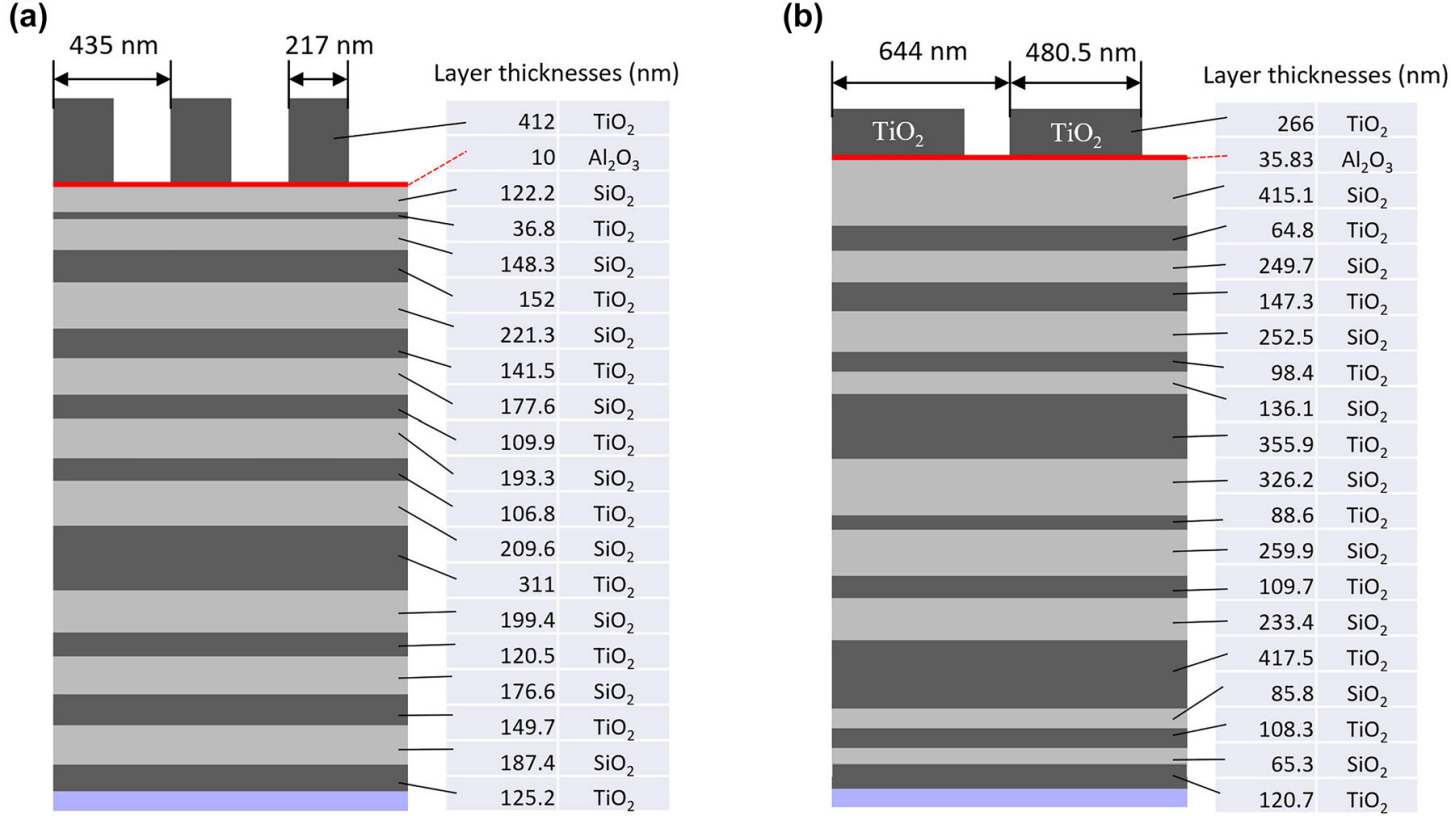
Optimized grating mirror design for (a) the pump and (b) output mirrors.

**Figure 4:**
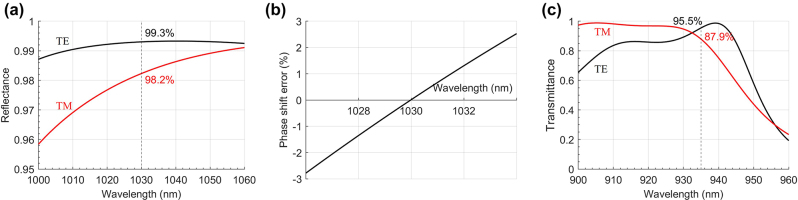
Calculated optical properties of the pump mirror. (a) Reflectance as a function of the incident light wavelength, with dashed line and annotation at the laser wavelength of 1030 nm. (b) TE and TM phase shift deviation from π around the laser oscillation wavelength. (c) Transmittance as a function of the incident light wavelength, with dashed line and annotation at the pump wavelength of 935 nm.

**Figure 5:**
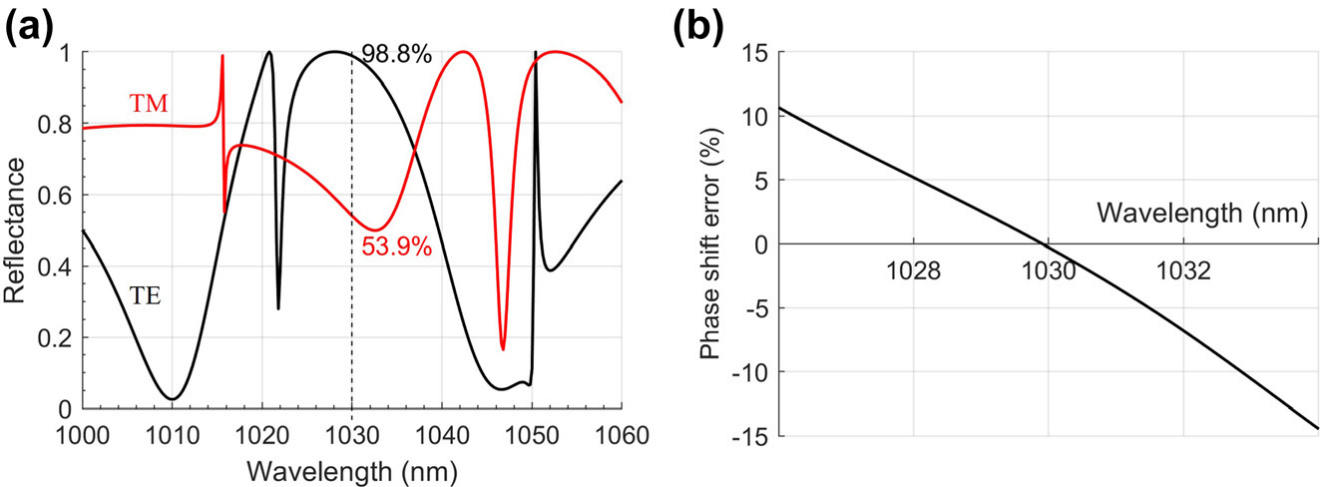
Calculated optical properties of the output mirror. (a) Reflectance as a function of the incident light wavelength, with dashed line and annotation at the laser wavelength of 1030 nm, and (b) TE and TM phase shift deviation from π of the output mirror.

The grating mirror design is based on the Fourier modal method, which is a method very well adapted to the scope of the problem, i.e., only stationary solutions to Maxwell equations are required and the inhomogeneous region has periodic boundary conditions in the lateral directions. It allows one to rigorously model the complete system of multilayer mirror and grating at the same time and it is also fast and accurate. For this paper, the software MCGrating [37] was used, since it is highly optimized and allows fast numerical parameter sweeps and optimizations. Details about the optimization procedure can be found in the Supplementary Materials. The best solutions for the pump and output mirrors found with this approach for the laser wavelength of *λ*_l_ = 1030 nm are shown in Figure 3. The spectral behavior of the reflectance and the TE/TM phase difference around the laser wavelength are shown in Figures 4 and 5 for the pump and output mirrors. The transmittance around the pump wavelength for the pump mirror is also shown in Figure 4(c).

## 4 Fabrication and characterization of the grating mirrors

### 4.1 Fabrication of the anisotropic mirrors

**Figure 6:**
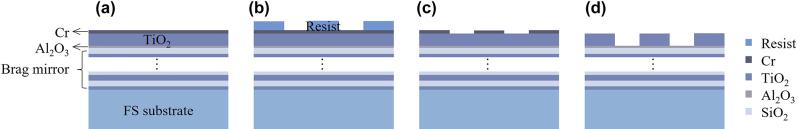
Schematic of the fabrication process. (a) Thin film deposition; (b) E-beam lithography; (c) ICP-RIE Cr etching and (d) ICP-RIE TiO_2_ etching and Cr removal.

**Figure 7:**
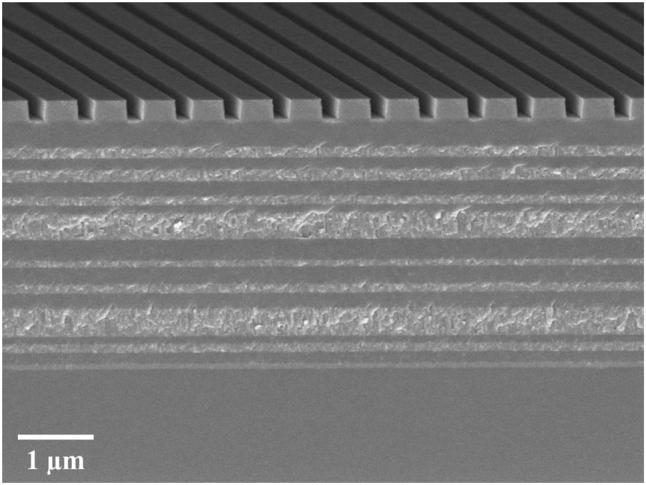
Scanning electron micrograph cross-section image of a representative output mirror.

The anisotropic mirrors consist of a Bragg multilayer with alternating TiO_2_ and SiO_2_ layers and a grating made of TiO_2_ on top, as shown in Figure 3. The multilayer coatings were prepared with thin film deposition techniques, while the TiO_2_ grating was fabricated by Electron-beam lithography and inductively coupled plasma reactive-ion etching (ICP-RIE), where a 50-nm-thick chromium (Cr) layer was used as an etching mask and a thin layer of Al_2_O_3_ served as an etch-stop layer for TiO_2_ etching. The different steps are outlined in Figure 6 and described in more detail in the Supplementary Materials. To achieve the required phase shift of π with as small an error as possible, ten grating patterns with slightly different linewidths but with the same period were prepared on one substrate. A scanning electron micrograph (SEM) cross-section of an output coupler is shown in Figure 7. Other SEM images are shown in the Supplementary Materials.

### 4.2 The ellipsometric characterization of the mirrors

The calculation of polarization eigenstates and eigenvalues requires the estimation of the parameters $R_{TE}\equiv {\left|r_{TE}\right|}^{2}$ , $R_{TM}\equiv {\left|r_{TM}\right|}^{2}$ and Δ for each mirror, cf. Eqs. S7 and S8 of Supplementary Materials. The general phase factor $\exp\left(i\varphi_{TE}\right)$ of each mirror is neither accessible nor important for that calculation. This can be done by using ellipsometry. A special ellipsometric setup, described in the Supplementary Materials, was designed to measure these optical properties at normal incidence and at the emission wavelength of the laser, *λ*_*l*_ = 1030 nm.

**Figure 8:**
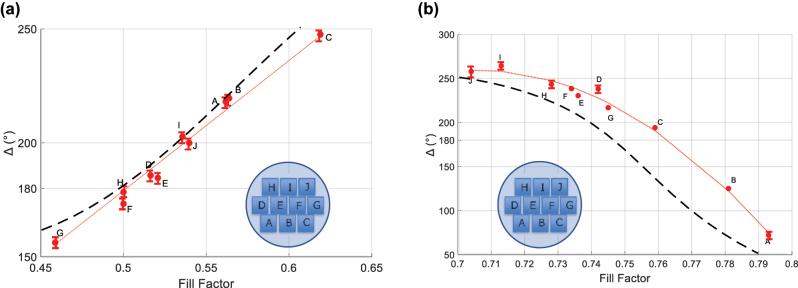
TE and TM phase shift of the (a) pump mirror and (b) output coupler in reflection as a function of the fill factor. The dashed lines are the corresponding simulation results and the thin dotted lines in red are a guide for the eye.

The measurement results are gathered for all pump mirrors in Table S1, and those of the output mirrors are shown in Tables S2 and S3 for reflection and transmission respectively in the Supplementary Materials. From Table S1, it appears that all the pump mirrors have high *R*_TE_ and *R*_TM_ reflectance values of similar magnitude at *λ*_l_ = 1030 nm, ranging from 92% to 97%, as desired. For both mirrors, measured differences in phase shift between different gratings can be traced to the different fill factors of the gratings, as can be seen in Figure 8(a) and (b) by the monotonous trend of the Δ values as a function of the fill factor. Also shown in dashed lines are the phase shift values predicted by numerical simulations. Pump mirror H has the phase shift (Δ = 178.4°) closest to the targeted 180° value. The transmission of the pump mirror at the pump wavelength of *λ* = 935 nm is about 91%. For the output mirror, from Table S2, the results vary greatly from one grating to another. Grating C is the one displaying the phase shift value (Δ = 188.8°) closest to the targeted 180° value. It also displays *R*_TE_ and *R*_TM_ reflectance of 89% and 48%, not too far from the specifications. Hence, most laser experiments were carried out with pump mirror H and output mirror C forming a standing wave resonator.

## 5 Laser experiments

The set-up used to carry out laser experiments was described in ref. [32] and is shown again for convenience in Figure S12 in the Supplementary Materials. The output mirror was mounted on a rotation stage to adjust the relative angle, *α*, between the two gratings’ principal axes. The diagnostic of the laser emission consisted in characterizing the polarization eigenvalues and eigenstates, the degree of polarization of the emission, the optical conversion efficiency, the beam quality and the emission spectrum as a function of the twist angle *α*.

### 5.1 Polarization eigenvalues and eigenstates, and other laser characteristics

The elements of the Jones matrix of each mirror were obtained from ellipsometric measurements, except for an unimportant (and inaccessible) general phase factor, cf. Section 4.2. From the knowledge of the Jones matrices of the two mirrors, *M*_1_ and *M*_2_, and their relative orientation, *α*, the Jones matrix of a round trip of the passive resonator, *J*_RT_, was obtained. The magnitude of the two eigenvalues, *ω*_1_ and *ω*_2_, of *J*_RT_ determines the round-trip losses of the two modes, while their different phase values determine their frequency difference.

**Figure 9:**
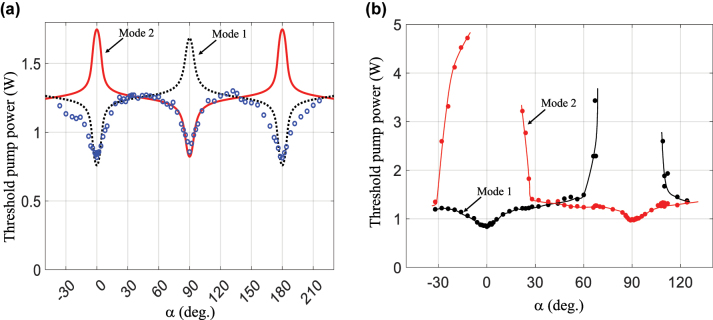
Measured and calculated threshold pump power values, *P*_th_. (a) Measured (circles) and calculated (dotted and solid curves for modes 1 and 2 respectively) *P*_th_ values of each polarization eigenmode as a function of twist angle α. (b) Similarly measured *P*_th_ values showing the threshold for dual polarization emission.

The mode with the lowest losses, i.e., with the largest magnitude of the eigenvalue, $\max\left\{\left|\omega_{i}\right|\right\}$ , determines the threshold of laser emission, which is the minimum pump power required to achieve laser oscillation, *P*_th_, proportional to (cf. Supplementary Materials, Eqs. S14–S17):

(1) $$P_{th}\left(\alpha \right)\propto N\sigma_{as}d-\ln\left(1-A\right)-2\ln\left(\max\left\{\left|\omega_{i}\left(\alpha \right)\right|\right\}\right),$$

where *N* is the Yb^3+^ doping concentration, *σ*_as_ is the effective absorption cross-section at the lasing wavelength, *d* is the thickness of the active material, and *A* is the distributed roundtrip loss. The first term on the right-hand side of Eq. (1) is known, the second term is assumed negligible, and the third term is the main contribution arising from the α-dependent eigenvalues calculated from *J*_RT_. Experimental values of *P*_th_ as a function of *α* are shown in Figure 9(a), together with the theoretical values calculated from Eq. (1) with only one adjustable scaling factor. Experimentally measured values of *P*_th_ follow the theoretical predictions well. As can be seen in Figure 9(a), the two modes have very different losses around *α* = 0° and 90°; hence, we expect that only one polarization eigenstate oscillates for *α* values in those neighborhoods. On the other hand, round-trip losses are very similar between *α* = 30° and 60°, as well as between *α* = 120° and 150°; dual polarization oscillation is thus expected for these *α* values.

If the pump power is increased well above *P*_th_, it is possible to detect the threshold of dual polarization emission. This happens when the round-trip losses of the weaker mode are not too large compared to those of the main mode. Dual polarization emission can then be easily observed by using an elliptical polarizer, made of a quarter waveplate and a linear polarizer. A proper adjustment of the orientation angle of each element will block a specific mode and partially transmits the other. Threshold of dual polarization emission is shown for modes 1 and 2 in Figure 9(b). As expected, dual polarization oscillation is well suppressed near *α* = 0° and 90° even at pump power values that exceed the threshold value by several times. As *α* departs from 0°, a second threshold eventually appears, first at very high pump power, and then decreases to reach values similar to the first threshold for *α* values around 45°; at that point, dual polarization emission takes place as soon as the threshold of oscillation is reached. It is noteworthy that the second threshold shown in Figure 9(b) is significantly higher than what Figure 9(a) suggests. This is due to the saturation of the gain medium during laser operation, which restraints the growth of the excited population, *N*_2_, as the pump power is increased. It was shown that, in the presence of gain saturation of a homogeneous active material, the gain of the competing modes does increase with pump power above threshold due to spatial hole burning [38], although much slower than below threshold.

**Figure 10:**
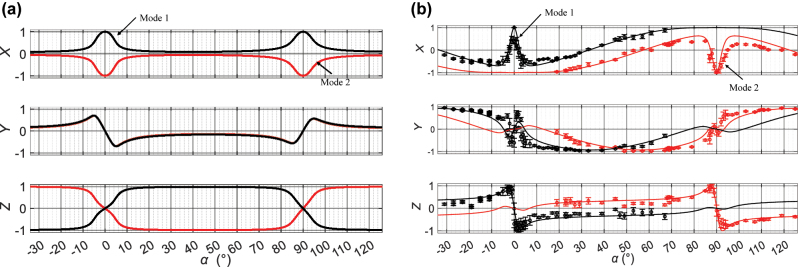
Poincaré coordinates of the polarization eigenstates. (a) Calculated coordinates (*x*, *y*, *z*) on the Poincaré sphere of the two polarization eigenstates *inside* the resonator. (b) Measured and calculated coordinates (*x*, *y*, *z*) of the two polarization eigenstates *outside* the resonator.

The polarization state of the two modes was then determined by measuring the angular positions of the quarter waveplate and the polarizer that achieve extinction in turn determine the orientation and eccentricity of the ellipse of polarization. The latter can be mapped on (*x*, *y*, *z*) coordinates of the Poincaré sphere, cf. Supplementary Materials. The calculated coordinates of the two polarization eigenstates inside the resonator for the wave travelling towards the output mirror are shown in Figure 10(a). The measured coordinates of the two eigenstates outside the resonator are shown in Figure 10(b). The knowledge of the Jones matrix of the output mirror in transmission, accessible from the data of Table S3, enables one to compare experimental with calculated values. Experimental polarization states agree well with those calculated from the experimentally measured Jones matrices, Figure 10(b). One can also see that the polarization state of the output beam, Figure 10(b), rapidly changes from nearly right circular at *α* ≈ −2.5° to the circular state of opposite chirality at *α* ≈ 2.5°, passing through horizontal polarization near *α* = 0°; a similar phenomenon takes place near *α* = 90°. Having a laser mode of well-defined chirality is a direct consequence of the resonator itself being chiral for *α* ≠ 0° and *α* ≠ 90°: taking a mirror image of the resonator is equivalent to changing *α* into −*α*; as a result, the beam’s chirality, both inside and outside the resonator, is also reversed and the (*x*, *y*, *z*) Poincaré coordinates are transformed into (*x*, −*y*, −*z*) when replacing *α* by −*α*, as shown in Supplementary Materials, Figure S13.

**Figure 11:**
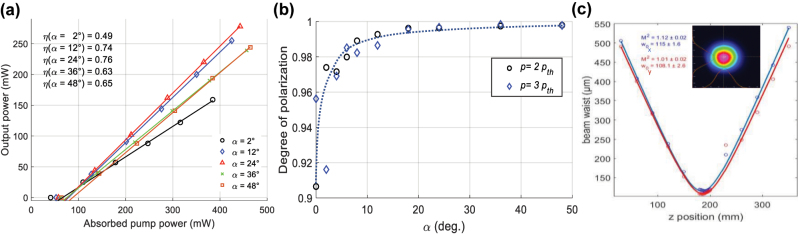
Measured slope efficiencies, degree of polarization and beam quality of the chiral laser. (a) Measured output power as a function of absorbed pump power for several *α* values.  For each α value, the slope efficiency is shown in the figure. (b) Measured degree of polarization as a function of *α*. (c) M^2^ measurements and beam profile obtained for *α *= 12^o^.

The output power as a function of absorbed pump power is shown in Figure 11(a) for several *α* values. The threshold of oscillation and the optical slope efficiency vary with the *α* value. As the latter is increased from 0°, the power leaking from the output mirror is increased, so the threshold increases, while the slope efficiency increases. Threshold pump power ranges between 40 and 70 mW while the slope efficiency with respect to the absorbed pump power ranges between 50% and 76%: it is highest in the region where multimode emission is best suppressed as shown in Section 5.2.

The degree of polarization, *p*, for various *α* values is shown in Figure 11(b). This was done by measuring the polarization extinction ratio, η, of the output beam. The parameters *p* and η are related by the equation: $p=\left(\eta -1\right)/\left(\eta +1\right)$ . We found *p* to be higher than 0.99, for most *α* values except near *α* = 0°, where it is still higher than 0.9. We note that the change of *p* value strongly correlates with the rapid change of the polarization state inside the resonator, from rectilinear to circular between *α* = 0° to 10°_,_ cf. Figure 10(a). This high degree of polarization value close to 1 validates our approach using a Jones matrix formalism.

The quality of the output beam was also measured for several *α* values; in all cases and for any pumping power, the *M*^2^ value was found to be very close to unity. An example of beam diameter measured as a function propagation distance for *α* = 12° is shown, together with a Gaussian beam profile, in Figure 11(c). This is the result of the good mode matching of the pump beam with the fundamental gaussian mode of our resonator. This rules out the existence of multiple transverse modes in laser operation.

### 5.2 The emission spectra

As shown in Figure 1(a), adjusting *α* makes it possible to control the contrast of the standing wave of the laser mode. At *α* angles different from 0° or 90°, the contrast is significantly reduced, and one would expect good suppression of multiple longitudinal mode emission arising from spatial hole burning in this region. The spectral separation between consecutive longitudinal modes of our laser is in the order 64 GHz, i.e., smaller than the resolution available with our spectrometers. Hence, we used a Fabry–Perot étalon (FPE) to separate the spectral components of the emitted beam. The free spectral range and finesse of the étalon, respectively FSR_FE_ = 200 GHz and *F* = 25, are large enough to easily identify consecutive modes of our laser without overlapping interference orders. Details of the experimental setup are shown in the Supplementary Materials, Figure S12.

**Figure 12:**
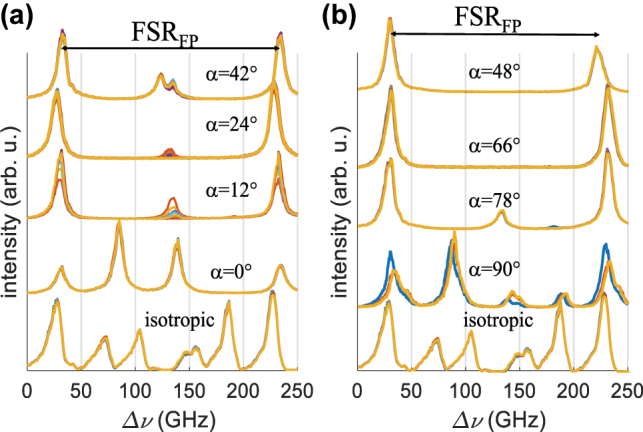
Emission spectra for ten CCD images for a standard resonator for various *α* values of the grating mirrors (a) from *α* = 0 to *α* = 42° (b) from *α* = 48° to *α* = 90°. The emission spectra for an identical laser using isotropic mirrors are shown at the bottom. The spectral lines repeat at every free spectral range of the Fabry–Perot, FSR_FP_. The spectra are vertically shifted for clarity.

Emission spectra are shown in Figure 12 for *α* values ranging from 0° to 42°, Figure 12(a), and from 48° to 90° in Figure 12(b). The emission spectrum of a similar laser using isotropic mirrors is shown for comparison at the bottom of each figure. For all the shown traces, the laser operated at twice the laser oscillation threshold. For each *α* value, ten different traces are shown, showing the reproducibility of the measured emission spectra. For the anisotropic resonator, the emission spectrum was found to change significantly as the twist angle *α* between the two mirrors was changed. For *α* = 0°, the grating lines of the two mirrors were aligned and this forced laser oscillation to take place in the linear TE polarization state, which has lower losses than TM-polarized light. However, multimode operation was found because the counterpropagating waves produced maximum interferential contrast resulting in spatial hole burning, like in a standard resonator. At *α* = 12°, the spectrum became bimodal and at *α* = 24°, the second mode almost disappears. At *α* = 42°, the spectrum became multimode again. At *α* = 48° and *α* = 66°, the measured spectra were single mode although, at the former angle *α* = 48°, the polarization state was found to occasionally hop from one polarization eigenstate to the other. The emission spectrum then became increasingly multimode as the *α* value approaches 90°. For a conventional resonator, the emission spectrum was found to contain several peaks inside one free spectral range of the FP étalon, cf. the bottom traces of Figure 12(a) and (b).

**Figure 13:**
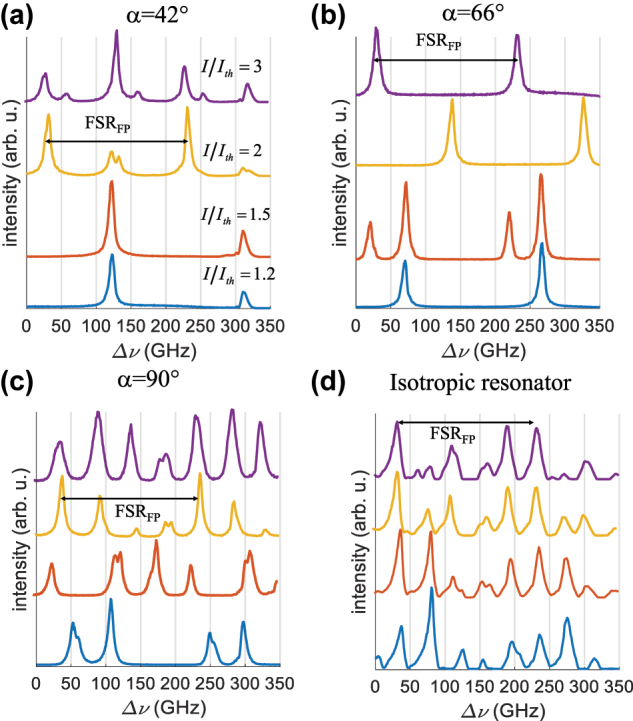
Evolution of the emission spectrum as a function of the pumping intensity for anisotropic resonators (a) *α* = 42°, (b) *α* = 66° and (c) *α* = 90°, and (d) for a resonator with isotropic mirrors for a pumping intensity *I* ranging from 1.2, 1.5, 2 and 3 times the threshold current intensity, *I*th, in the diode laser.

The evolution of emission spectra as a function of the pump power is shown in Figure 13 for the case of (a) *α* = 42°, (b) *α* = 66°, (c) *α* = 90° and (d) a resonator of similar geometry with conventional isotropic mirrors. The current intensity in the pump laser diode was increased from 1.2 to 3 times the threshold value. The emission remained mostly single mode for *α* = 66°, while for *α* = 42°, the emission became mainly bimodal from a pumping intensity equal to twice the threshold value. For the anisotropic resonator with *α* = 90°, the number of modes increased from 3 to 5, while for an isotropic resonator, the number of frequencies increased from 5 to 6 for the same pumping range. The highly multimode operation observed near *α* = 0° and *α* = 90° and the improvement of the single mode character between these two values were consistent with the quasi cancellation of axial hole burning phenomenon away from *α* = 0° and *α* = 90°, as seen in Figure 1(a), curve B. Also, the coexistence of polarization states, which is more frequently seen around 42° and 48° than at *α* values closer to *α* = 0° and *α* = 90°, was consistent with the discrimination predicted by calculations shown in Figure 9(a) and confirmed by the measurements of the second threshold in Figure 9(b).

## 6 Summary and conclusion

Nanostructured laser mirrors made of a diffraction grating engraved on the top layer of a Bragg mirror were designed to work close to a PT-symmetric laser in the polarization space, i.e., with a phase shift close to π and different reflection coefficients for TE- and TM-reflected polarization states of the output coupler at normal incidence. These mirrors were fabricated by depositing a Bragg multilayer and then by writing a diffraction grating in the top layer using electron-beam patterning and reactive ion etching. A laser was then built by forming a resonator with these mirrors and by end pumping a Yb^3+^-doped YAG ceramics placed between them.

The emission characteristics of such laser were measured at various values of the twist angle, *α*, between the two mirrors’ principal axes. The evolution of the threshold pump power and the polarization eigenstates as a function of *α* were found to be consistent with calculations made from the measured optical characteristics of each mirror. In addition, by slightly changing *α*, large changes in the polarization state of the emitted beam, spanning circular polarization states of opposite chiralities, were obtained, adding flexibility to the available emission properties of such laser. The efficiency was found to increase in the *α* region where the emission spectrum was single mode. The degree of polarization was found to be very high, although less so near *α* = 0°. The *M*^2^ measurements were found to be consistent with single mode emission in the fundamental Gaussian mode for all the experimental conditions. Operating a microchip laser with anisotropic mirrors enabled a significant reduction of the number of emitted modes in comparison to a standard design using isotropic laser mirrors. Single, or quasi-single mode emission, was enabled with *α* values in the order of *α* ≈ 16°–24° or *α* ≈ 66°–74°.

These anisotropic mirrors eliminate the need for intracavity elements, leading to shorter resonator lengths, which further promote emission into a single longitudinal mode by producing larger spacing between consecutive modes. The resonator length can be reduced to the thickness of the active material by designing resonant grating laser mirrors directly fabricated at the two surfaces of a platelet of active material. This design would enhance the mechanical robustness of the device and would also allow efficient water cooling, also allowing the measurement of its spectral characteristics at even higher power. Such miniature single mode laser with high temporal coherence is compatible with planar processing of the semiconductor industry and is an attractive alternative to other competing laser devices, such as VCSELs, which cannot be easily power-scaled, or with semiconductor lasers based on BIC lasers or topological insulator laser, for applications in photonic integrated circuits as well as LiDARs for the automotive applications or remote sensing.

## Supplementary Material

Supplementary Material Details
