# Supplementary material for: A chiral microchip laser using anisotropic grating mirrors for single mode emission
Source: Nanophotonics. 2023 Mar 24;12(9):1741–52. doi: 10.1515/nanoph-2022-0783 (PMC11502084; doi:10.1515/nanoph-2022-0783)
Supplement: Supplementary file 1 — Supplementary Material Details [file j_nanoph-2022-0783_suppl_001.pdf]

Supplementary Materials

A chiral microchip laser using anisotropic grating mirrors for single mode emission

Fangfang Li[1], Shawn Lapointe[2], Théo Courval[2], Marina Fetisova[1], Thomas Kämpfe[3], Isabelle Verrier[3], Yves Jourlin[3], Petri Karvinen[1], Markku Kuittinen[1], Jean-François Bisson[2]*

[1]*Center for Photonics Sciences, University of Eastern Finland, P.O. Box 111, FI-80101 Joensuu, Finland*
[2]*Département de physique et d'astronomie, Université de Moncton, 18 Antonine-Maillet Ave., Moncton, Canada, E1A 3E9*
[3]*Univ Lyon, UJM-Saint-Etienne, CNRS, Institut d'Optique Graduate School, Laboratoire Hubert Curien UMR 5516, F-42023, Saint-Etienne, France*

*jean-francois.bisson@umoncton.ca

Table of contents

1. Design of the grating mirrors
2. Fabrication of the grating mirrors
3. Optical characterization of the mirrors
   a. The optical characterization of the multilayers and the grating mirrors
   b. Ellipsometric characterization of the grating mirrors
4. Laser experiments
   a. Polarization eigenstate analysis
   b. Experimental setup
   c. Connection between the eigenvalues and the pump power at threshold
   d. Demonstration of dual polarization emission

1. Design of the grating mirrors

The problem in finding a grating mirror that fulfills the conditions outlined in Fig. 2 and Table I is that there is no known method that provides a direct solution to the inverse problem, i.e., to derive the structure parameter from the desired optical characteristics via an algorithmic approach. The Fourier Modal Method, as well as other methods, only permits to find the optical response of a given geometrical configuration of multilayer and grating. Due to the large number of parameters, i.e., more than ten layer thicknesses and refractive indices, grating height, duty cycle, period, refractive index, an exhaustive parameter search is not feasible. Therefore, a mixed approach is applied, using analytically derived starting configurations, followed by numerical optimization, whereby the specific approach is slightly different for the pump and output mirrors.

For the pump mirror, the starting point is a multilayer system that produces a strong reflection for light under normal incidence at the desired laser wavelength of λ=1030 nm and high transmission at the pump wavelength of λ=935 nm for a large enough angular range. This is achieved by using a $\lambda/4$ stack of high and low refractive index materials. It is basically a bandpass, with the band-edge positioned between the pump and laser wavelengths. Without grating and due to normal incidence, there is no distinction between TE and TM polarizations at this point. Then the grating is added. The period, duty cycle and height are chosen to allow a coupling to a waveguide, formed by the grating itself and the topmost layer, as explained for example in Ref. [S1]. Since the absolute value of the dephasing of TE and TM reflections with respect to the incident light does not have any physical significance for the application, only the phase difference between TE and TM is considered. The grating has to fulfill fabrication-related limitations, which shall be given here as follows:

- a period shorter than 711 nm to avoid propagating higher orders that will guide the light out of the multilayer and the laser in undesired paths;
- a period larger than 300 nm as a limitation of the grating fabrication process;
- a grating height smaller than 500 nm to avoid too deep grooves that are hard to etch;
- a linewidth between 0.2 and 0.8 of the period to avoid unstable or difficult-to-fabricate grating shapes, i.e. very thin lines or grooves.

The grating fabrication is an analogous process with a considerable remaining uncertainty in the grating parameters. A perfect design is therefore not practical if it is too narrow-band, considering the performance variation with changing grating parameters. Grating waveguide resonance effects can be either extremely narrow (e.g., Ref. [S2]) or broadband (e.g., Ref. [S3]). In our case, the transmission and reflection of TE and TM need to be decisive, whereas a slower performance variation is required for wavelength and grating parameters changes, allowing one to choose a working point adapted to fabrication tolerances.

To further tackle fabrication process uncertainties, a parameter-variation approach is chosen, i.e. deliberate variation of the most critical grating parameters over multiple gratings on one wafer and subsequent selection of the best suited element based on its actual optical response. This approach has to be already included in the design process. The most uncertain grating parameter for the chosen lithographic fabrication method is the duty cycle, which depends on the specific processing conditions during resist exposure, development, and the subsequent etching of the uppermost grating layer. Very

well controllable on the other hand are the grating period, defined by e-beam writing, and the grating depth, using an etch-stop layer. Hence, the duty cycle is varied on the wafer by varying the linewidth at fixed value of the period. In the design, the goal is therefore to find a grating mirror configuration with a stable, but slowly and monotonically varying TE/TM phase shift around a medium duty-cycle value. As for the period and grating height, we look for the most stable behavior around the specified values. A starting point for a grating is found by varying one of the grating parameters, while looking at the reflection spectra in TE and TM and the phase shifts. Several parameter combinations exist that result in comparable characteristics.

Varying all the multilayer mirror layer thicknesses allows one to significantly optimize the optical response of the element, due to intricate changes in the waveguide coupling coefficients for TE and TM polarization. However, it is not feasible to analytically reason about the underlying physical mechanisms of these changes, so numerical optimization is used. It is based on the Davidon-Fletcher-Powell method, which is a quasi-Newton method, also known as the Variable Metrics method. It optimizes a system by multidimensional, systematic parameter variation for minimizing a merit function, following a steepest gradient path. The merit function is designed to represent all aspects previously discussed: it comprises the reflection of TE and TM polarized light and the phase difference, at different wavelengths around the design wavelength, with adaptable weight factors. The spectral width of the merit function has to be chosen to allow very good performance at the grating mirror nominal parameters, but avoid the appearance of too sharp resonances, which could destroy the desired functionality for slight non-normal conditions. A sufficient spectral width of the grating mirror's operating point usually also creates tolerance in other variations, like incidence angle and grating mirror parameters, which is necessary for a proper functioning in the laser cavity. Such additional constraints can also be explicitly added to the merit function to further fine-tune the solution. In the numerical optimization, free parameters have to be defined. We follow an approach where we gradually increase the number of grating mirror parameters allowed to vary, starting with the grating and the top-most layers, and then gradually descending down the multilayer stack, to finally allow all layer thicknesses and the grating to be varied. This gradual approach avoids divergent behavior of the optimization towards not useful, e.g., layer thicknesses tending to 0, or unpractical, e.g., extremely thick layers parameter combinations.

Fig. S1 shows the angular performance around the laser and pump wavelengths for the pump mirror with the parameters given in Fig.3(a). An analysis of the gratings' performance for linewidth and grating height variations is shown in Fig. S2 and S3 for the same mirror. One can see that a grating height deviation can be compensated by a shift of the linewidth due to the monotonously increasing phase shift. The design of the output mirror follows the same guidelines as the pump mirror, but the grating parameters are adjusted to meet the specifications shown in Fig. 1 due to the fabrication error in multilayer mirror coating. The performance of the output mirror, in analogy to the previous figures for the pump mirror, is shown in Fig. S4 to S6.

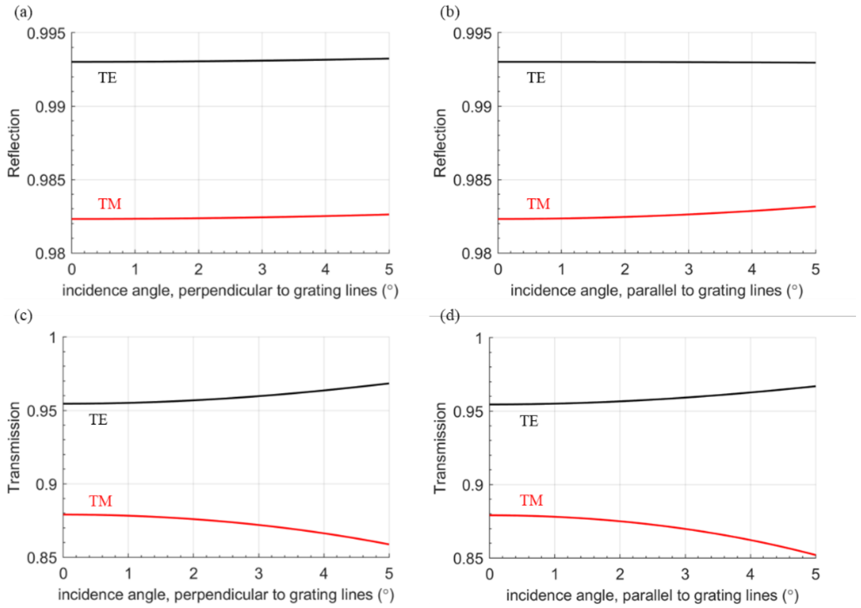

Fig. S1. Reflection at 1030 nm wavelength of the pump mirror as a function of the incident angle with incident light (a) perpendicular to grating lines and (b) parallel to grating lines. Transmission at 935 nm wavelength for the pump mirror with incident light (c) perpendicular and (d) parallel to grating lines.

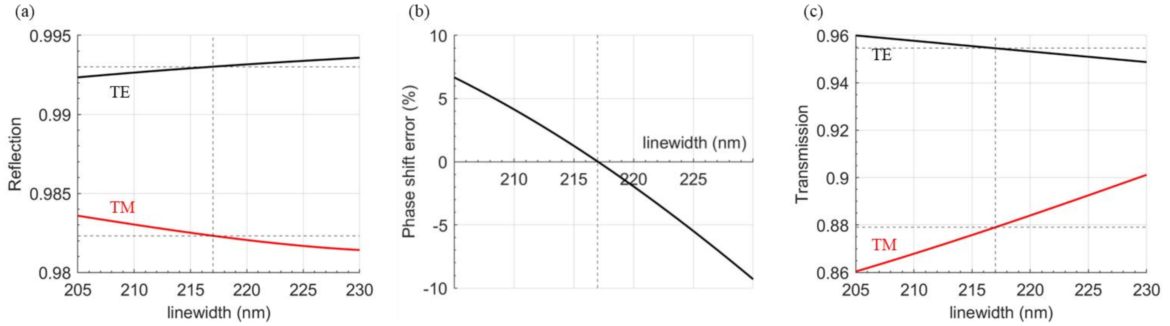

Fig. S2. Grating mirror performance of the pump mirror for grating linewidth variation. (a) Reflection at 1030 nm wavelength, (b) TE and TM phase shift deviation from π at 1030 nm wavelength, and (c) transmission at 935 nm wavelength.

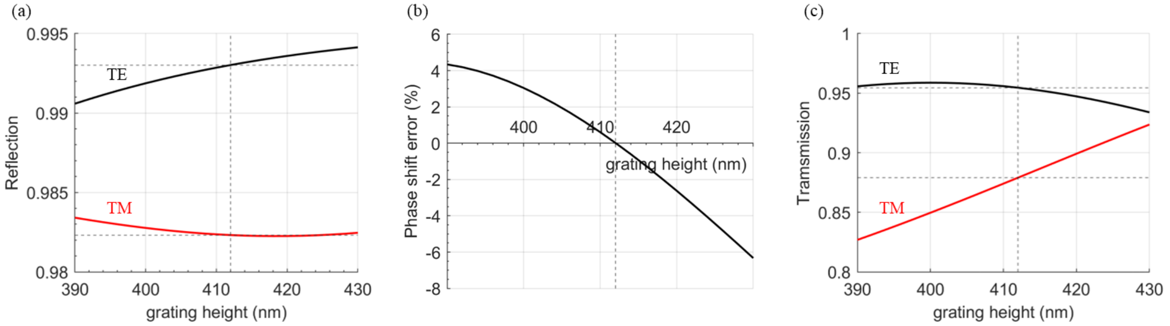

Fig. S3. Grating mirror performance of the pump mirror for grating height variation. (a) Reflection at 1030 nm wavelength, (b) TE and TM phase shift deviation from π at 1030 nm wavelength, and (c) transmission at 935 nm wavelength.

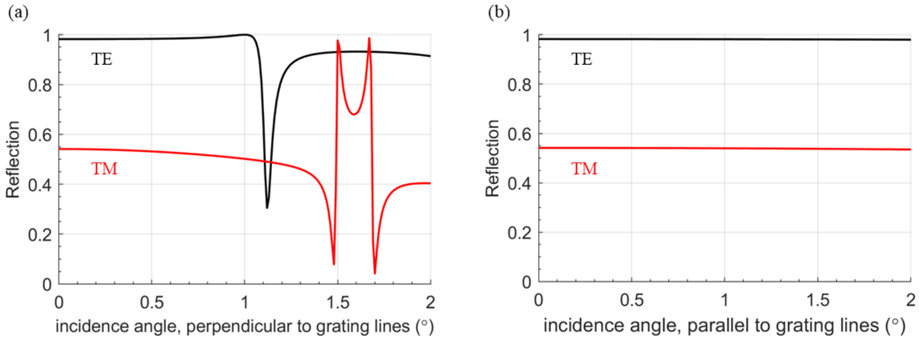

Fig. S4. Reflection of the output mirror as a function of the incident angle at the laser wavelength 1030 nm, with incident light (a) perpendicular to grating lines and (b) parallel to grating lines.

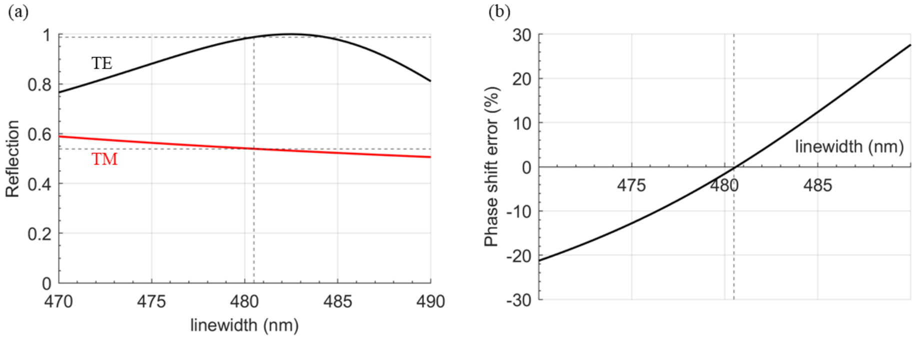

Fig. S5. Output grating mirror performance for grating linewidth variation. (a) Reflection and (b) TE and TM phase shift deviation from π at the laser wavelength 1030 nm.

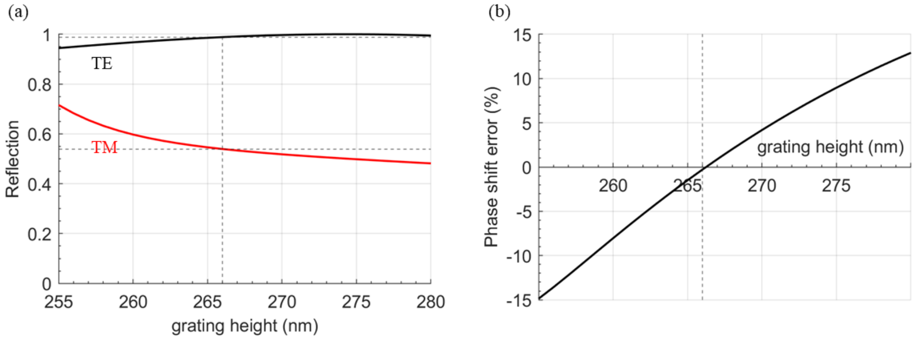

Fig. S6. Output mirror performance for grating height variation. (a) Reflection and (b) TE and TM phase shift deviation from π at the laser wavelength 1030 nm.

2. Fabrication of the grating mirrors

Both the pump and output mirrors were fabricated on 3-mm-thick, 1”-diameter fused silica substrates. The grating fabrication process steps are illustrated in Fig. S7. The layers in Fig. S7(a) were coated with different methods. The multilayer Bragg mirror, consisting of alternating TiO_2/SiO_2 layers, was customized by a commercial supplier (Oplatek Group Oy, Finland). They deposited the layers using e-beam evaporation with in-situ optical monitors. The rest of the layers were prepared at the University of Eastern Finland. A thin layer of Al_2O_3 served as an etch-stop layer to prevent over etch into the Bragg mirror during TiO_2 etching, while a 50-nm-thick chromium (Cr) layer was used as the etching mask in TiO_2 grating etching. The Al_2O_3 and TiO_2 layers on top of the Bragg mirror were coated by thermal atomic layer deposition (ALD) at 120°C using system Beneq TFS 200, with deposition rates of 0.12 nm/cycle and 0.06 nm/cycle, respectively. The precursor used for Al_2O_3 deposition was Trimethylaluminium (TMA), and $TiCl_4$ for TiO_2. Magnetron sputter was employed for Cr coating using sputter coater Quorum Q300T T Plus. Thickness of the Cr layer was controlled by the sputter time, and the deposition rate was approximately 20 nm/min.

After multilayer thin film deposition, electron-beam lithography (EBL) was used to pattern the gratings. First, O_2 plasma was applied on the sample for 1 minute to remove possible moisture. Second, a ~250-nm-thick layer of E-beam resist AR-P 6200 (Allresist GmbH, Germany) was spin-coated on the sample. Then, the resist was patterned using EBL system Raith EBPG 5000+ ES. To achieve a phase shift as close to π as possible, 10 grating patterns with slightly different duty-cycle were exposed on one sample at the same time. Each of the patterns had a size of 5×5 mm^2, with 50 μm between adjacent patterns. The sample was then developed with the following steps: soaked in developer ethyl 3-ethoxypropionate for 60 s and then 30 s in isopropanol, followed by rinsing with ultrapure water and blow drying with N_2. The sample profile after development is depicted in Fig. S7(b).

Inductively coupled plasma-reactive ion etching (ICP-RIE) was utilized to transfer patterns from electron-beam resist to Cr and subsequently to TiO_2. Cr was etched with Cl_2/O_2 gas mixture using Oxford Plasmalab 100, and 2 minutes of O_2 plasma was applied afterward to remove the resist residues, cf. Fig. S7(c). Then C_4F_8/O_2 gases were used to etch TiO_2 in Oxford Plasmalab 80 Plus. Finally, the Cr mask was removed again by ICP Cr etching, cf. Fig. S7(d).

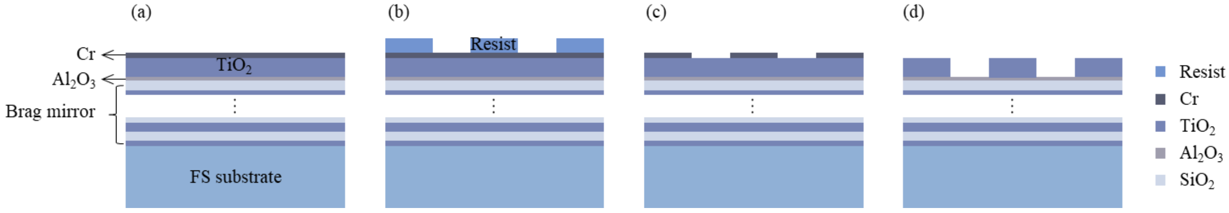

Fig. S7. Schematic of the fabrication process. (a) thin film deposition; (b) EBL patterning; (c) ICP-RIE Cr etching; (d) ICP-RIE TiO_2 etching and Cr removal.

The depths of the TiO_2 etching were measured by Dektak 150 stylus profiler equipped with a 12.5-µm tip, which were 396 nm and 258 nm for the pump and output mirrors respectively. Note that the TiO_2 depth was measured from the fabricated identifiers that were 40 µm wide rather than from the grating itself. In addition, the duty-cycle of the patterns was inspected by scanning electron microscopy (SEM) (Zeiss LEO 1550), with 7 nm of sputtered Cr as a conductive layer. Examples of SEM images of the pump and output mirrors are shown in Fig. S8.

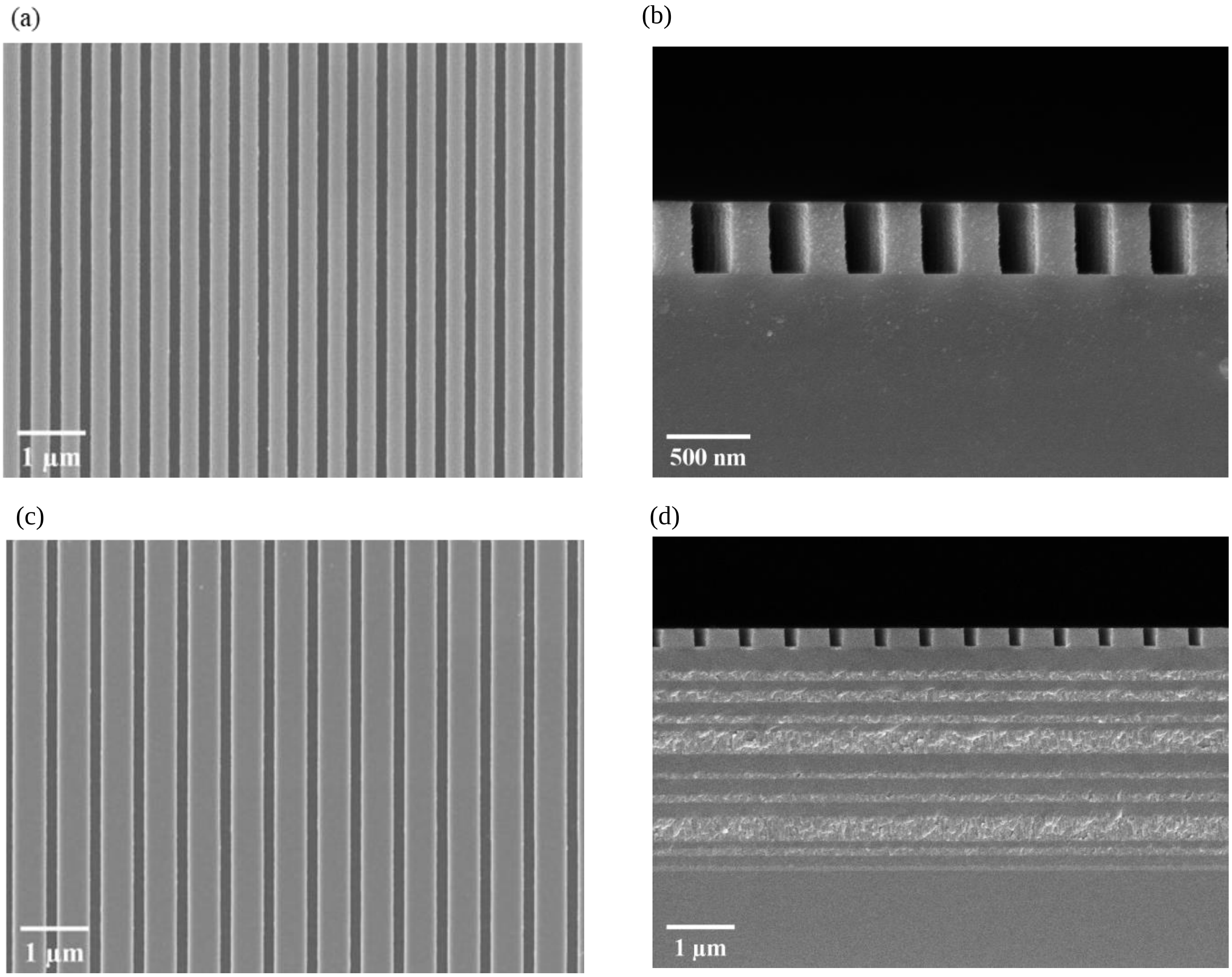

Fig. S8. Top view and cross-section SEM images of the (a,b) pump and (c,d) output mirrors. In (b), the test grating was on a substrate without multilayer.

3. Optical characterization of the mirrors

a. The optical characterization of the multilayers and the grating mirrors

Transmission spectra of the pump and the output mirrors, measured with the spectrophotometer PerkinElmer LAMBDA 1050+, before and after coating the top Al_2O_3 and TiO_2 layers by ALD, but

before fabricating the gratings, are shown in Fig. S9. The measured transmittance spectra are found to be in good agreement with the calculated spectra using the values shown in Fig. 3. Transmission and reflection spectra after the gratings were deposited are shown in Fig. S10.

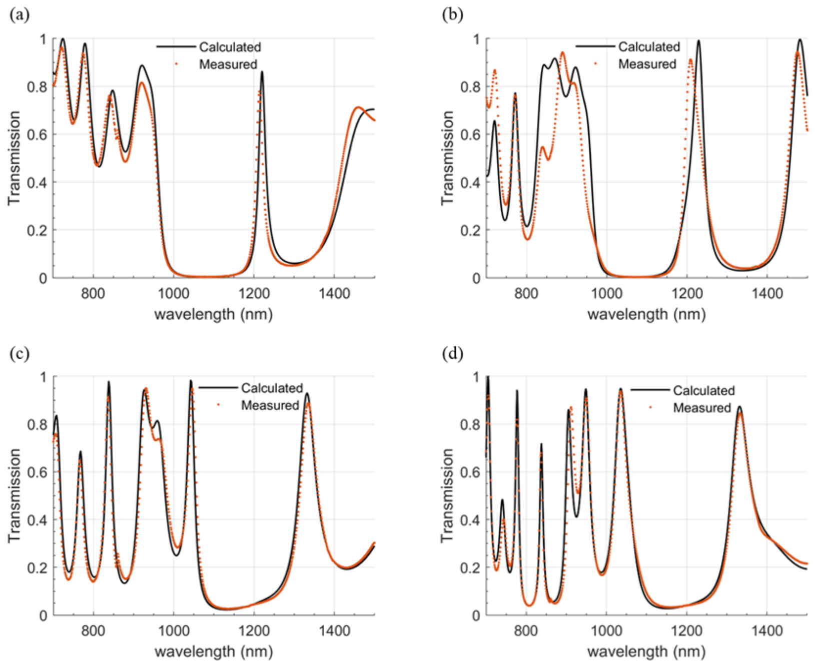

Fig. S9. Calculated and measured transmission of the pump mirror with (a) only evaporated multilayer coatings and (b) with both evaporated layers and ALD top layers. Similar plots are shown for the output mirror in (c) and (d).

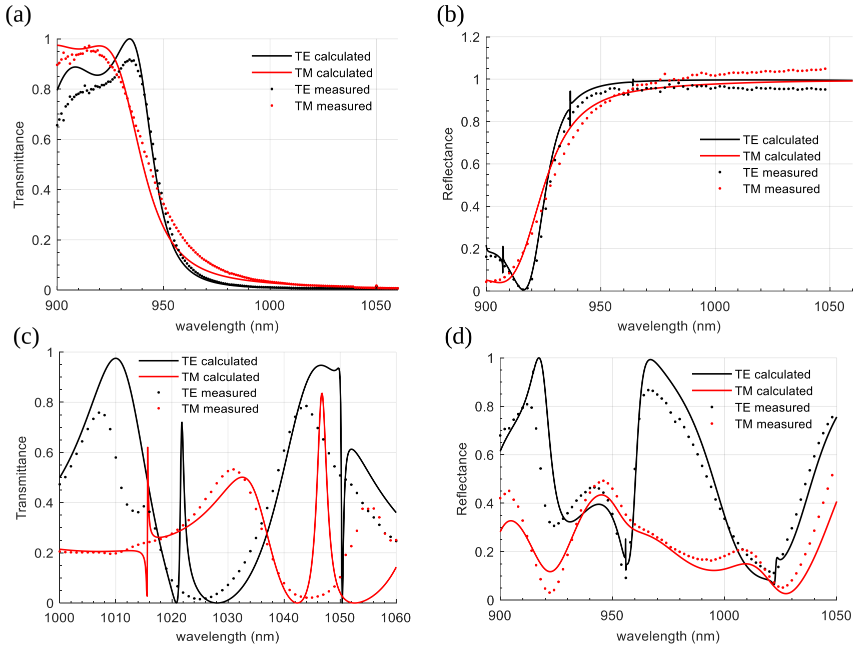

Fig. S10. Calculated and measured transmission at normal incidence and reflection at 20° incidence angle of the pump mirror with grating (a-b). Similar plots are shown for the output mirror in (c) and (d).

b. Ellipsometric characterization of the grating mirrors

Optical ellipsometry at normal incidence of the fabricated grating mirrors was used in order to determine the reflectance of the TE and TM polarizations as well as their relative phase shift *Δ*. The optical setup, shown in Fig. S11, is described as follows. A custom Yb^{3+}: $Y_3Al_5O_{12}$ (YAG) laser emitting at λ=1030 nm was built, and the laser was passed through a linear polarizer with its transmission axis oriented at a fixed angle of $\theta_1 = 45°$. Half of the diagonally polarized incident light is then reflected off a non polarizing beam splitter (BS) and sent to a reference silicon photodetector D_1, while the other half of the beam power is transmitted by the BS. In reflection mode, the incident light is reflected by the sample, i.e., the pump or output mirror, at normal incidence and then reflected off the BS and passed through another polarizer P_2 and sent towards a second silicon photodetector D_2. Three photo-signal measurements are taken with D_2, with the P_2 polarizer oriented at $\theta_2 = 0°$, 45° and 90°.

Prior to measuring the mirrors' properties, a calibration procedure is carried out, wherein the ratio of the photo-signal voltage values read by the two detectors V_2/V_1 is measured for an isotropic aluminum mirror standard of known reflectance R=0.94, for these three angles of the polarizer P_2. The intensity ratio is proportional to I, where [S4]:

$$I(\theta_2) = \frac{1}{2}\left(S_0 + S_1\cos(2\theta_2) + S_2\sin(2\theta_2)\right). \qquad \text{(S1)}$$

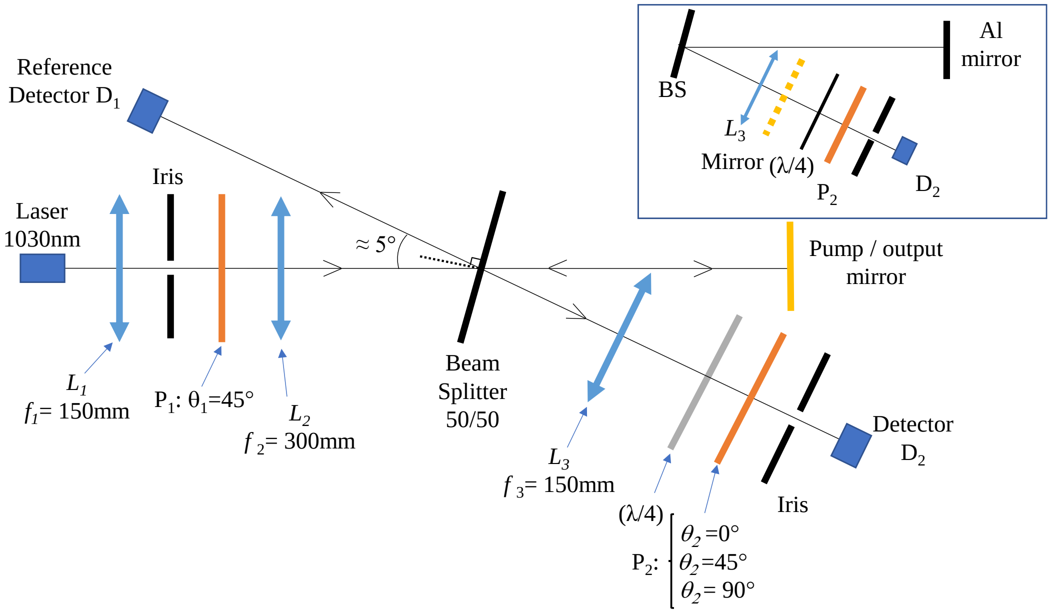

Fig. S11. Experimental set-up for ellipsometric measurements of the grating mirrors at normal incidence at 1030 nm. Measurements are performed both without and with the compensator (λ/4).

Here, S_0, S_1, and S_2 are the first three elements of the Stokes vector, where $\vec{S} = [S_0, S_1, S_2, S_3]^T$. For non-depolarizing mirrors whose Jones matrices are diagonal and for incident light linearly polarized at 45°, we have:

$$S_0 = \frac{R_{TE} + R_{TM}}{2}, \qquad \text{(S2a)}$$

$$S_1 = \frac{R_{TE} - R_{TM}}{2}, \qquad \text{(S2b)}$$

$$S_2 = \sqrt{R_{TE} R_{TM}} \cos\Delta \,. \qquad \text{(S2c)}$$

For the aluminum mirror, for which $R_{TE} = R_{TM} = R$ and cos(Δ)=1, we obtain $I(\theta_2=0°) = R/2$, $I(\theta_2=45°) = R$ and $I(\theta_2=90°) = R/2$. These measurements enable the determination of three proportionality constants, which are calibration coefficients, $\eta(\theta_2=0)$, $\eta(\theta_2=45)$ and $\eta(\theta_2=90)$, given by:

$$\eta(\theta_2) = \left.\frac{I(\theta_2)}{V_2/V_1}\right|_{\text{Aluminum mirror}} \qquad \text{(S3a)}$$

The estimated values of η are then used to determine ellipsometric coefficients $I(\theta_2)$ of the samples to be measured, from the measured ratios V_2/V_1 using the equation:

$$I(\theta_2) = \eta(\theta_2) V_2/V_1 \qquad \text{(S3b)}$$

Coefficients R_{TE}, R_{TM}, and cos(Δ) are then deduced from Eqs. (S2).

Now, the determination of cos Δ only enables one to estimate the absolute value of Δ. In order to determine its sign, we repeat the calibration procedure and measurement by adding a quarter waveplate with horizontal fast axis in front of the polarizer P_2, as shown in Fig. S10. Then, the intensity ratio V_2/V_1 as a function of θ_2 is proportional to I, where [S4]

$$I(\theta_2) = \frac{1}{2}\left(S_0 + S_1 \cos(2\theta_2) - S_3 \sin(2\theta_2)\right), \qquad \text{(S4)}$$

where, for diagonally polarized incident light and non-depolarizing material:

$$S_3 = \sqrt{R_{TE} R_{TM}} \sin\Delta \;. \qquad \text{(S5)}$$

In the calibration step with the compensator with an aluminium mirror as a standard, because Δ=0, one obtains $I(\theta_2=0°) = I(\theta_2=45°) = I(\theta_2=90°) = R/2$, which enables the determination of three calibration coefficients $\eta(\theta_2=0)$, $\eta(\theta_2=45)$ and $\eta(\theta_2=90)$. Then $I(\theta_2=0°)$, $I(\theta_2=45°)$ and $I(\theta_2=90°)$ of the unknown grating samples are measured and, from Eqs. S2a), S2b), S4 and S5, R_{TE}, R_{TM}, and sin(Δ) can be estimated. The combination of both sets of measurements, with and without compensator, makes it possible not only to dispel the ambiguity on the sign of Δ but it also enables a consistency check of the two measurements by calculating $\cos^2\Delta+\sin^2\Delta$ from the two measurements, which should be very close to unity. As shown in Tables S1 to S3 the two Δ estimates, called respectively Δ-cos and Δ-sin, are in most case quite consistent. There is third estimate in these figures, Δ-tan, which is obtained from the tangent and the knowledge of the signs of cos Δ and sin Δ.

Finally, the measurements were also performed in transmission for the output coupler. This required only changing the position of the mirror, i.e., placing it in front of the second polarizer and placing an aluminium mirror at the same place as where the reflection measurements are performed (cf. inset of Fig. S11). These transmission parameters were useful for the determination of the polarization state inside the resonator composed of the pump and output mirrors from measurements taken at the output of the resonator. The results obtained by ellipsometry are summarized in Table S1 for the pump mirrors and in Tables S2 and S3 for the output mirrors. Not shown in Table S1 is the measured transmittance at the pump wavelength of λ = 935 nm, in the order of 91 %. The observed evolution of the phase shift with fill factor of the gratings is shown in Fig. 7 of the main manuscript.

It is important to note that the mirror must be non-depolarizing in order that the Jones formalism and the use of eqs. (S2) and (S5) for describing the optical response of these mirrors be valid. This can be checked from the estimated values of the four Stokes coefficients. The degree of polarization, *P*, is given by [S4]:

$$P = \sqrt{S_1^{\,2} + S_2^{\,2} + S_3^{\,2}} \Big/ S_0 \ . \qquad \text{(S6a)}$$

The degree of polarization is also obtained from the extinction ratio $\eta \equiv P_{\max} / P_{\min}$, given by:

$$P = (\eta - 1)/(\eta + 1) \, . \qquad \text{(S6b)}$$

From the measurements shown in Tables S1 and S2, *P* is found to be very close to 100% for the pump mirror H and is in the order of 98 % for the output mirror C used in our laser experiments. These values validate the Jones formalism and the use of Eq. (S2) and (S5) for describing the optical response of these mirrors.

Table S1. Reflection measurements of the pump mirrors. All gratings have the same period of 435 nm and differ only by their linewidth. Grating H, highlighted, was selected for laser experiments because of its most suitable phase shift and reflection coefficients.

	gratings	A	B	C	D	E	F	G	H	I	J
	Linewidth (nm)	241	243	268	221	226	220	200	216	233	231
Reflection data	**cos Δ**	**-0.783**	**-0.788**	**-0.384**	**-0.993**	**-0.996**	**-0.988**	**-0.907**	**-0.999**	**-0.926**	**-0.938**
	R_{TE}	0.962	0.943	0.932	0.966	0.969	0.971	0.985	0.972	0.983	0.954
	R_{TM}	0.973	0.951	0.969	0.941	0.934	0.931	0.925	0.934	0.967	0.919
	**sin Δ**	**-0.617**	**-0.637**	**-0.924**	**-0.100**	**-0.081**	**0.117**	**0.403**	**0.028**	**-0.387**	**-0.342**
	R_{TE}	1.050	0.933	0.929	0.954	0.958	0.946	0.985	0.965	0.980	0.945
	R_{TM}	1.064	0.944	0.955	0.928	0.923	0.932	0.921	0.924	0.963	0.911
	**cos²(Δ) + sin²(Δ)**	**0.9938**	**1.0272**	**1.0012**	**0.9969**	**0.9983**	**0.9901**	**0.9839**	**0.9988**	**1.0070**	**0.9957**
	Δ-cos(°)	218.46	217.98	247.45	186.57	185.21	171.18	155.03	177.45	202.20	200.36
	Δ-sin(°)	218.09	219.58	247.54	185.74	184.64	173.30	156.25	178.38	202.77	199.98
	Δ-tan(°)	218.23	218.95	247.46	185.75	184.64	173.26	156.05	178.38	202.68	200.02

Table S2. Reflection measurements of the output mirrors. All gratings have the same period of 644 nm and differ only by their linewidth. Grating C, highlighted, was selected for laser experiments because of its more suitable phase shift and reflection coefficients.

	gratings	A	B	C	D	E	F	G	H	I	J
	linewidth	505	499	485	466	469	471	474	463	452	444
Reflection data	**cos Δ**	**0.305**	**-0.576**	**-0.969**	**-0.525**	**-0.635**	**-0.521**	**-0.800**	**-0.447**	**-0.095**	**-0.208**
	R_{TE}	0.269	0.656	0.892	0.652	0.699	0.598	0.791	0.575	0.461	0.503
	R_{TM}	0.299	0.442	0.482	0.409	0.508	0.507	0.549	0.485	0.709	0.893
	**sin Δ**	**0.886**	**0.794**	**-0.150**	**-0.817**	**-0.744**	**-0.835**	**-0.587**	**-0.870**	**-0.972**	**-0.965**
	R_{TE}	0.281	0.646	0.877	0.640	0.687	0.593	0.779	0.565	0.459	0.497
	R_{TM}	0.296	0.438	0.486	0.404	0.503	0.499	0.548	0.475	0.699	0.879
	**cos²(Δ) + sin²(Δ)**	**0.8788**	**0.9626**	**0.9620**	**0.9431**	**0.9575**	**0.9691**	**0.9844**	**0.9561**	**0.9545**	**0.9750**
	Δ-cos(°)	72.22	125.19	194.23	238.34	230.57	238.57	216.91	243.46	264.56	257.98
	Δ-sin(°)	62.41	127.44	188.60	234.79	228.11	236.61	215.98	240.43	256.50	254.84
	Δ-tan(°)	70.99	125.97	188.77	237.28	229.53	238.01	216.30	242.81	264.43	257.82

Table S3. Transmission measurements of the output mirrors.

	gratings	A	B	C	D	E	F	G	H	I	J
Transmission data	**cos Δ**	**0.950**	**0.941**	**-0.586**	**-0.404**	**-0.235**	**-0.287**	**-0.483**	**-0.366**	**-0.537**	**-0.472**
	T_{TE}	0.671	0.253	0.059	0.273	0.237	0.292	0.209	0.356	0.497	0.316
	T_{TM}	0.621	0.535	0.554	0.509	0.365	0.362	0.422	0.464	0.213	0.124
	**sin Δ**	**-0.277**	**0.178**	**-0.806**	**-0.885**	**-0.956**	**-0.928**	**-0.839**	**-0.899**	**-0.780**	**-0.615**
	T_{TE}	0.663	0.260	0.059	0.271	0.234	0.290	0.208	0.342	0.481	0.307
	T_{TM}	0.635	0.543	0.571	0.529	0.371	0.367	0.439	0.485	0.228	0.131
	**cos²(Δ) + sin²(Δ)**	**0.9791**	**0.9176**	**0.9926**	**0.9473**	**0.9696**	**0.9443**	**0.9369**	**0.9412**	**0.8968**	**0.6014**
	Δ-cos(°)	341.78	379.73	234.15	246.17	256.42	253.32	241.10	248.55	237.50	241.82
	Δ-sin(°)	343.90	370.24	233.70	242.30	253.00	248.18	237.00	243.97	231.24	217.96
	Δ-tan(°)	343.73	370.69	233.99	245.47	256.21	252.82	240.05	247.86	235.43	232.49

4. Laser experiments

a. Polarization eigenstate analysis

The polarization eigenstate analysis of a resonator equipped with two anisotropic mirrors is done by calculating the Jones matrix of the round-trip inside such a resonator. We define the Jones matrix of the pump and output mirrors, M_1 and M_2 as:

$$M_1 = \begin{pmatrix} r_{1,TE} & 0 \\ 0 & r_{1,TM} \end{pmatrix}_{\{x_1,y_1\}} = \exp\left(i\varphi_{1,TE}\right)\begin{pmatrix} |r_{1,TE}| & 0 \\ 0 & -|r_{1,TM}|\exp\left(i\Delta_1\right) \end{pmatrix}_{\{x_1,y_1\}} \quad \text{(S7a)}$$

and

$$M_2 = \begin{pmatrix} r_{2,TE} & 0 \\ 0 & r_{2,TM} \end{pmatrix}_{\{x_2,y_2\}} = \exp\left(i\varphi_{2,TE}\right)\begin{pmatrix} |r_{2,TE}| & 0 \\ 0 & -|r_{2,TM}|\exp\left(i\Delta_2\right) \end{pmatrix}_{\{x_2,y_2\}}, \quad \text{(S7b)}$$

in their respective basis $\{x_1,y_1\}$ and $\{x_2, y_2\}$ of their principal axes, cf. Fig. 2, where

$$\Delta_1 \equiv \varphi_{1,TM} - \varphi_{1,TE} \tag{S8a}$$

and

$$\Delta_2 \equiv \varphi_{2,TM} - \varphi_{2,TE} \,. \tag{S8b}$$

The propagation matrix, P, also enters the calculation of the round-trip Jones matrix. For isotropic laser materials with negligible birefringence, either thermally-induced or intrinsic, it is proportional to the identity matrix:

$$P = \exp(i\phi + gd)\begin{pmatrix} 1 & 0 \\ 0 & 1 \end{pmatrix}, \tag{S9}$$

where

$$\phi = \frac{2\pi}{\lambda}\left(L + (n-1)d\right) \tag{S10}$$

is the accumulated phase shift in a single pass of length L and g is the amplitude gain per unit length inside the active medium of refractive index n and thickness, d. In a reference frame $\{x, y\}$ that bisects the principal axes $\{x_1, y_1\}$ and $\{x_2, y_2\}$ of the two mirrors, cf. Fig. 2 (left), the round-trip Jones matrix is calculated as:

$$J_{RT}(\alpha) = P \times TM_2T \times P \times TM_1T \,, \tag{S11}$$

where

$$T = \begin{pmatrix} \cos(\alpha/2) & \sin(\alpha/2) \\ -\sin(\alpha/2) & \cos(\alpha/2) \end{pmatrix} \tag{S12}$$

is the change-of-basis matrix from $\{x_1, y_1\}$ or $\{x_2, y_2\}$ to $\{x, y\}$ coordinate frame, cf. Fig. 2. Note that the J_{RT} matrix depends on the relative orientation α of the two mirrors' principal axes.

b. Experimental setup

The set-up used to carry out laser experiments was described in ref. [S5] and is shown again for convenience in Fig. S12. The output mirror was mounted on a rotation stage to adjust the relative angle, α, between the two gratings' principal axes. A 1-mm-thick, 10%-doped Yb-doped YAG ceramics (Baikowski Japan Corp.) was end-pumped with a fiber-coupled laser diode (K940FA3RN-30W model from BWT Beijing Ltd.) delivering up to 30W from a 105-µm-diameter, NA = 0.22 core and emitting at λ_p = 935 nm, mode-matched to the fundamental TEM_{00} Gaussian mode with a pair of spherical lenses to suppress high-order transverse modes. The laser operates in quasi-continuous-wave (QCW) regime with 10-ms pump pulses and 10% duty cycles. In these experiments, the mirrors were in contact with the active elements and therefore the fluctuations of the two mirrors' positions were correlated. This allowed a reduction of mode hopping compared to mirrors being physically separated from each other.

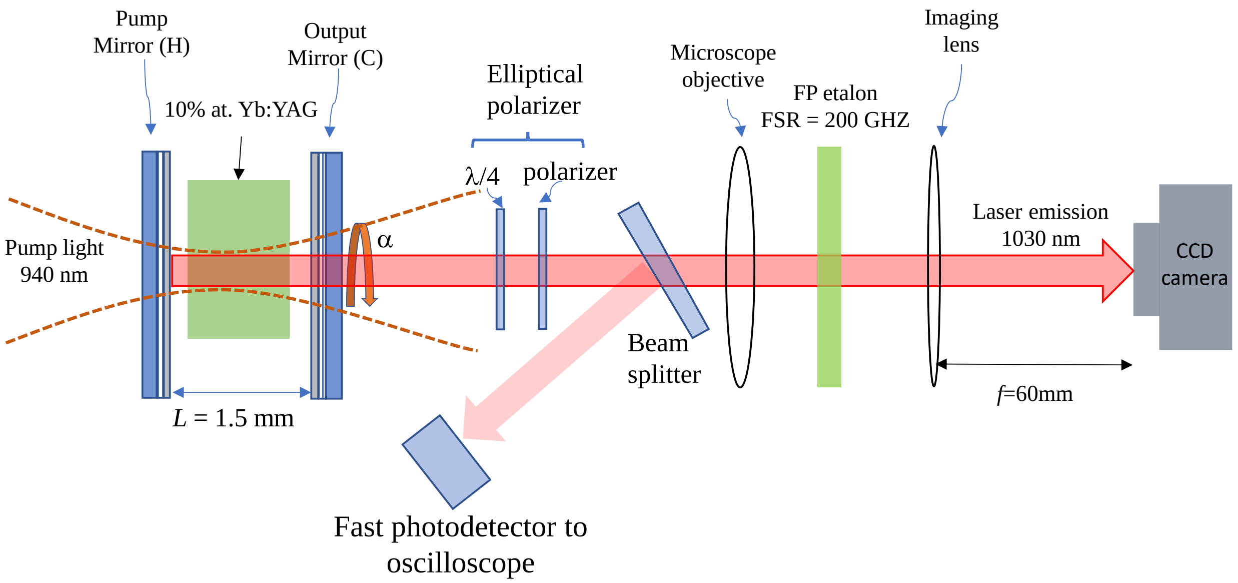

Fig. S12 Laser setup and the diagnostics tools used for the determination of the polarization state and the emission spectrum.

The diagnostic of the laser emission mainly consisted in characterizing the polarization eigenstates and the emission spectrum as a function of the α angle. For the determination of the polarization state outside the resonator, the emitted radiation is transmitted through a quarter wave plate (the compensator) followed by a polarizer, called an analyzer, each mounted on a rotation stage that allowed us to adjust the rotation angle in order to reach as close an extinction of the transmitted beam as possible. Extinction can be obtained first by converting the generally elliptical beam into a rectilinear polarization by aligning the compensator's fast axis with one axis of the elliptical pattern of the transverse electric field vector and then by seeking extinction by rotating the analyzer. The angle of the fast axis of the compensator directly produced so-called angle ξ and the angle of the analyzer at extinction was subtracted from ξ to produce the angle χ. Then, the *x*, *y*, *z* coordinates on the Poincaré coordinates were determined by using the following formulas [S7]:

$$\begin{pmatrix} x \\ y \\ z \end{pmatrix} = \begin{pmatrix} \cos(2\xi)\cos(2\chi) \\ \sin(2\xi)\cos(2\chi) \\ -\sin(2\chi) \end{pmatrix}. \tag{S13}$$

The chiral nature of the resonator is shown in Fig. S13, illustrating the fact that the mirror image of a twisted resonator is equivalent to changing the sign of α. When doing so, the elliptical polarization state also undergoes mirror reflection, which reverses the sign of both ξ and χ angles, which in turn changes the sign of *y* and *z* Poincaré coordinates.

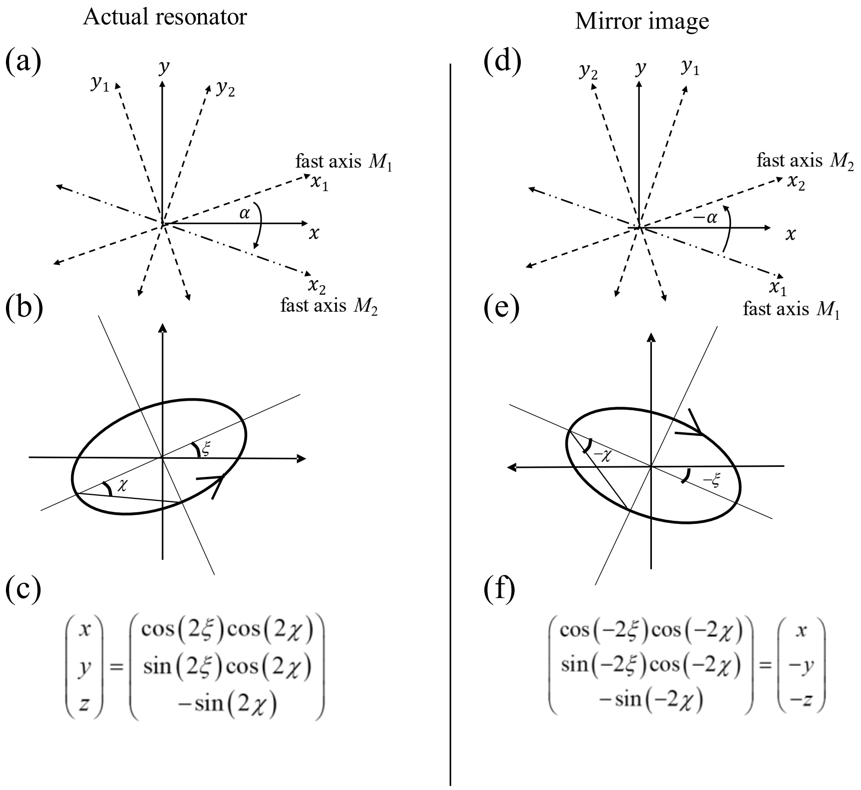

Fig. S13 The two mirrors' principal axes, twisted by angle α (a), produce elliptical polarization eigenstate (b), whose ellipticity χ and inclination ξ can be converted into Poincaré coordinates (c). The transformation of the resonator (d) and polarization state (e) by a mirror plane is equivalent to changing *α* by -*α* and the resulting Poincaré coordinates [17] are converted from (*x*,*y*,*z*) to (*x*,-*y*,-*z*), (f), which reverses the chirality of the polarization state.

For the determination of the emission spectrum, both fiber-coupled spectrum analyzer with a grating monochromator (Ocean Optics model USB2000+) and a Fabry-Perot interferometer and were used. The former enabled the determination of the emission on a large spectral bandwidth but with limited resolution in the order of δλ= 1 nm, as shown in Fig. S14 as a function of α. The later enabled high enough resolution to resolve individual mode frequencies but with limited free spectral range (FSR). The laser beam was focused into a Faby-Perot étalon (FPE) with finesse F = 25 and FSR = 200 GHZ. Each emitted frequency from the laser was then converted into a set of interference rings corresponding to different orders of interference inside the FPE. The rings can be visualized on a CCD camera placed at the focal distance of a focusing lens. The FSR is chosen significantly larger than the FSR of the laser resonator, in the order of 64 GHz, so that the apparition of consecutive longitudinal modes in the emission spectrum can easily be interpreted from a duplication of the interference rings.

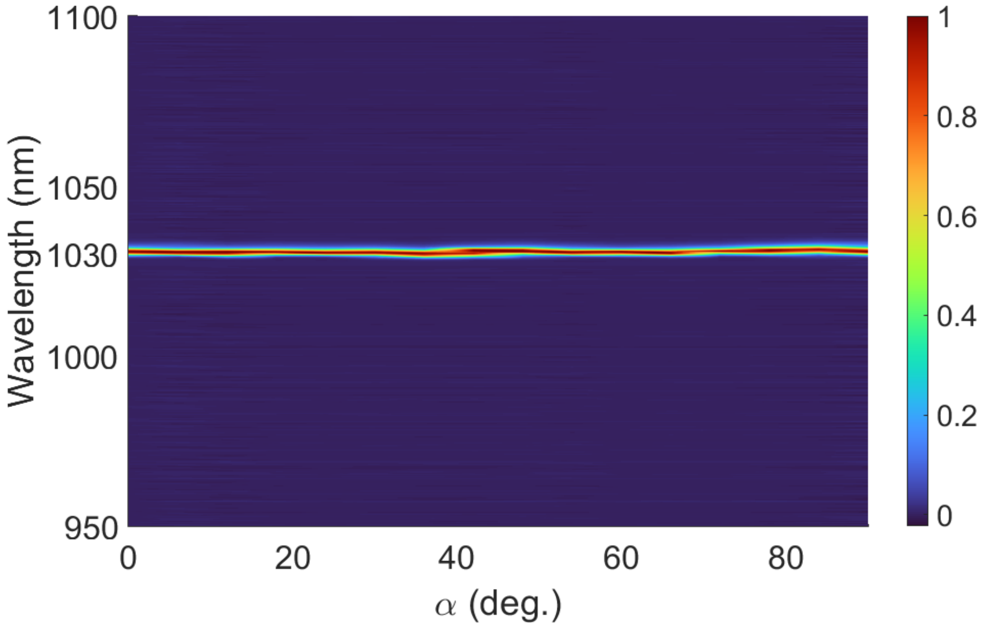

Fig. S14. Normalized emission spectrum of the laser as a function of α. The linewidth is limited by the resolution of the spectrometer and it is resolved by using a Fabry-Pérot étalon.

c. Connection between the eigenvalues and the pump power at threshold

As the absorbed pump power is increased, the population density, N_2, of the upper Stark level $^2F_{5/2}$ of the laser transition of Yb^{3+} increases linearly until the corresponding reduction of the population density, N_1, of the ground Stark level, $^2F_{7/2}$, becomes significant, at which point saturation of absorption sets in. This happens at power density values in the order of the saturation intensity parameter I_{sat}, which at a pump wavelength λ_p=935 nm, is in the order of I_{sat}=20 kW/cm^2 [S6]. This is much larger than the pump intensity I measured at threshold, in the order of 1 kW/cm^2. Hence, N_2 scales linearly P, with a proportionality constant that depends on the absorbed fraction of the incident power, the beam diameter, the absorption cross-section and the fluorescence lifetime of the excited level. The population N_2 in turn determines the gain per unit length, g, inside the active medium. At the oscillation threshold, the round-trip gain exactly compensates the round-trip losses, which arise mostly from the reflection of the mirrors and from fractional round trip loss sources, A, distributed inside the resonator. The threshold condition reads:

$$\max\left\{\left|\omega_i\right|^2\right\}\left(1-A\right)\exp\left(2gd\right)=1, \tag{S14}$$

where $\left|\omega_i\right|$ is the magnitude of the complex eigenvalues of the Jones matrix of the round-trip inside the resonator, d is the thickness of the active medium, the gain per unit length, g, is given by:

$$g = N_2\sigma_{es} - N_1\sigma_{as} = N_2\left(\sigma_{es}+\sigma_{as}\right) - N\sigma_{as}, \tag{S15}$$

where $N = N_1+N_2$ is the Yb^{3+} concentration, and σ_{es} and σ_{as} are the effective emission and absorption cross-sections of Yb^{3+} at the laser wavelength. From (S14-S15), one obtains the excited-state population at threshold, given by:

$$N_2 = \frac{N\sigma_{as}d - \ln(1-A) - 2\ln\left(\max\left\{|\omega_i|\right\}\right)}{2d\left(\sigma_{es}+\sigma_{as}\right)} \qquad \text{(S16)}$$

Since the pump power scales linearly with N_2, P_{th} is thus proportional to:

$$P_{th}(\alpha) \propto N\sigma_{as}d - \ln(1-A) - 2\ln\left(\max\left\{|\omega_i(\alpha)|\right\}\right), \qquad \text{(S17)}$$

which is eq. (1) of the main manuscript.

d. Demonstration of dual polarization emission

When dual polarization emission takes place, such as around 45°, it is possible to isolate one polarization eigenstate by passing the light through a suitably adjusted elliptical polarizer to eliminate one mode and keep only the other eigenmode, and vice versa. This is shown for α=42° in Fig. S15. We also observed the dual polarization emission in time. We found that the polarisation modes do not coexist but jump from one to the other during the emission. By placing the elliptical polariser on the path of the photodiode, we can block one of the polarization modes on the photodiode while still seeing the full spectral content on the CCD camera. This allows us to see on the oscilloscope the effects of blocking one mode, as shown in Fig. S16. A single mode operation in one polarization eigenstate or the other is seen in the top and middle interference patterns, while a pair of rings due to the dual polarization emission is shown in the bottom pattern of Fig. 16(a). Each interference ring can be converted to a frequency, Fig. S16(b) [S5]. When blocking one eigenstate on the photodiode by using an elliptical polarizer, nothing is seen (top trace), the full power of one mode is received (middle trace), or the transition from one mode to the other (bottom trace) are seen, Fig. S16(c).

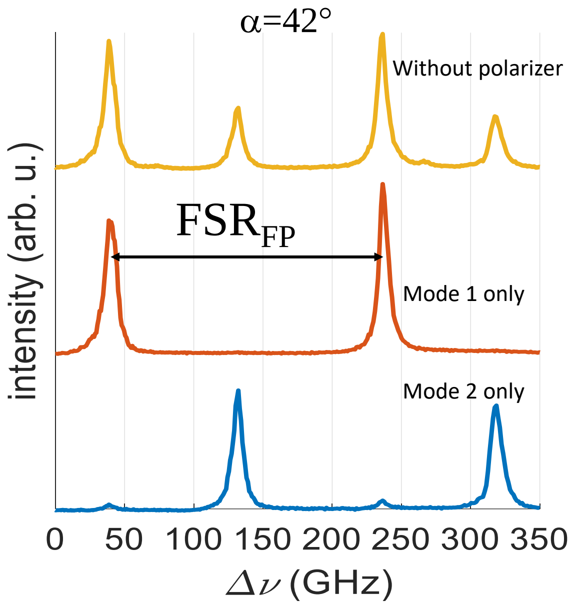

Fig. S15. Emission spectrum observed at α=42° without polarizer (top row) and with an elliptical polarizer that only transmits first and second polarization eigenmode (middle and bottom rows) at laser diode intensity of 2.5 times the threshold value.

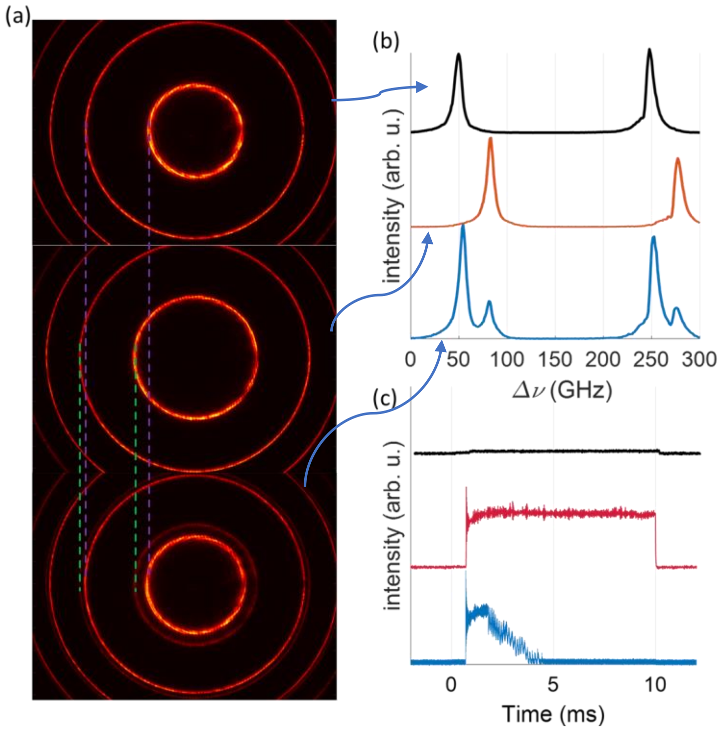

Fig. S16. Emission spectra observed at α = 42°. (a) CCD camera images of the Fabry Perot interference rings at different times; (b) corresponding analysis of the rings to obtain the spectral content; (c) oscilloscope traces of the photodiode intensity; for the latter, an elliptical polarizer is adjusted to suppress one polarization eigenmode on the path of the photodiode.

References

[S1] Manuel Flury, Alexandre V. Tishchenko, and Olivier Parriaux, “The Leaky Mode Resonance Condition Ensures 100% Diffraction Efficiency of Mirror-Based Resonant Gratings,” J. Lightwave Technol. **25**, 1870-1878 (2007)

[S2] O. Parriaux, T. Kämpfe, F. Garet, and J. L. Coutaz. “Narrow band, large angular width resonant reflection from a periodic high index grid at terahertz frequency,” Optics Express, **20**, 28070-28081 (2012).

[S3] Y. Jourlin, S. Tonchev, A.V. Tishchenko, F. Lacour, and O. Parriaux, “Resonant-grating reflection extended to wide-band, large-aperture beams by waveguide-mode coalescence,” Opt. Express **20**, 29155-29163 (2012).

[S4] B. Schaefer, E. Collett, R. Smyth, ‘‘Measuring the Stokes polarization parameters’’, Am. J. Phys. **75** 163-168 (2007).

[S5] J. F. Bisson, K. N. Amouzou, ‘‘Elimination of spatial hole burning in solid-state lasers using nanostructured thin films’’, Appl. Opt. **59(5)**, A83-A91 (2020).

[S6] D. Kouznetsov, J.-F. Bisson, K. Takaichi, K.-I. Ueda, ‘‘High-power single-mode solid-state laser with short, wide instable cavity,’’ J. Opt. Soc. Am. B **22(8)**, 1065-1619 (2005).

[S7] J.-F. Bisson, K. N. Amouzou, ‘‘Controlling spatial hole burning in lasers using anisotropic laser mirrors’’, J. Opt. Soc. Am. B **36(12)**, 3322-3332 (2019).

End of the Supplementary Materials.
